# Metagenomic and metabolomic analysis of changes in intestinal contents of rainbow trout (*Oncorhynchus mykiss*) infected with infectious hematopoietic necrosis virus at different culture water temperatures

**DOI:** 10.3389/fmicb.2023.1275649

**Published:** 2023-10-16

**Authors:** Qiang Hai, Jianfu Wang, Weiguo Kang, Shuru Cheng, Jie Li, Nana Lyu, Yajun Li, Zhiyuan Luo, Zhe Liu

**Affiliations:** College of Animal Science and Technology, Gansu Agricultural University, Lanzhou, China

**Keywords:** rainbow trout, IHN, temperature, metagenomic, metabolomic

## Abstract

Infectious hematopoietic necrosis (IHN) is a major disease that limits the culture of rainbow trout. In practical production, it has been found that the temperature of the culture water is a crucial factor affecting its mortality. Currently, little is known about how temperature affects the immune response of rainbow trout gut microbiota and metabolites to IHNV. In this study, our main objective is to analyze the changes in gut microorganisms of rainbow trout (juvenile fish with a consistent genetic background) after 14 days of infection with IHNV (5 × 10^5^ pfu/fish) at 12–13°C (C: injected with saline, A: injected with IHNV) and 16–17°C (D: injected with saline, B: injected with IHNV) using metagenomic and metabolomic analyses, and to screen for probiotics that are effective against IHNV. The results showed that infection with IHNV at 12–13°C caused Eukaryote loss. Compared to Group C, Group A showed a significant increase in harmful pathogens, such as Yersiniaceae, and a significant alteration of 4,087 gut metabolites. Compared to group D, group B showed a significant increase in the abundance of Streptococcaceae and *Lactococcus lactis*, along with significant changes in 4,259 intestinal metabolites. Compared with their respective groups, the levels of two immune-related metabolites, 1-Octadecanoyl-glycero-3-phosphoethanolamine and L-Glutamate, were significantly upregulated in groups A and B. Compared to group B, Group A showed significantly higher pathogenic bacteria including *Aeromonas*, *Pseudomonas*, and Yersiniaceae, while group B showed a significant increase in Streptococcaceae and *Lactococcus lactis*. Additionally, there were 4,018 significantly different metabolites between the two groups. Interestingly, 1-Octadecanoyl-sn-glycero-3-phosphoethanolamine and L-Glutamate were significantly higher in group A than in group B. Some of the different metabolites in C vs. A are correlated with *Fomitopsis pinicola*, while in D vs. B they were correlated with *Lactococcus raffinolactis*, and in A vs. B they were correlated with *Hypsizygus marmoreus*. This study exposed how rainbow trout gut microbiota and metabolites respond to IHNV at different temperatures, and screens beneficial bacteria with potential resistance to IHN, providing new insights and scientific basis for the prevention and treatment of IHN.

## 1. Introduction

Rainbow trout (*Oncorhynchus mykiss*) is a cold-water fish with significant economic value that is widely farmed worldwide. However, the massive outbreaks of infectious hematopoietic necrosis (IHN) resulted in a large number of rainbow trout deaths, which seriously constrained the healthy development of rainbow trout farming industry. Infectious hematopoietic necrosis virus (IHNV) belongs to the genus *Novirhabdovirus* in the Rhabdoviridae family, consisting of a bullet-like particle approximately 150–190 nm in length and 65–75 nm in diameter, which encloses a nodeless, negative-sense single-stranded RNA genome with approximately 11 kb (Morzunov et al., 1995; Dixon et al., 2016). IHNV is the causative agent of Infectious hematopoietic necrosis (IHN), which can lead to significant mortality in salmon stocks (Abbadi et al., 2021). The disease has been classified as a notifiable animal disease by the World Organization for Animal Health (OIE) and a category Π animal disease by the Ministry of Agriculture and Rural Development in China (Anderson et al., 2008). When IHN occurs, diseased fish show phenotypic features such as abdominal distention, protruding eyes and darkened skin, as well as extensive necrosis of major hematopoietic organs (Yong et al., 2019). IHNV can also replicate in large numbers in the intestine and cause necrosis of the intestinal wall cells in rainbow trout. The main characteristic lesion is yellow mucus in the intestine, with mucus-like, tubular feces hanging from the anus (Huang et al., 2022), which can cause mortality rates of up to 80–100% in young rainbow trout (Louboutin et al., 2021; Ren et al., 2022). Since its first report in the United States, the disease has spread widely in Canada, Europe, Japan and China, causing serious economic losses to the aquaculture industry (Kim et al., 2023). Nowadays, the prevention and control of IHN is mainly based on the development of vaccine. However, to date, no effective IHN control measures have been identified. In production, it has been found that temperature is an important factor influencing the disease of IHN, when the culture water temperature is between 3°C and 15°C, IHN can cause a large number of deaths, and the disease stops when the culture water temperature exceeds 15°C (Dixon et al., 2016; Wargo et al., 2017).

The intestine is the body’s closest contact with the external environment and is not only a digestive organ but also the largest immune organ in the body (Jin et al., 2023). Microorganisms in the gut influence the health of the host and play an important role in maintaining a healthy gut and resisting pathogenic invasion (Den Besten et al., 2013; el Aidy et al., 2013). When a viral agent invades a host, it can cause changes in its gut microbes and their metabolites and metabolite derivatives (Dong et al., 2019; Wu et al., 2021). It was found that rainbow trout infected with IHNV for 14 days had a significantly higher abundance of some beneficial bacteria such as Bacillus in their intestines (Huang et al., 2022). In addition, some bacterium isolated from the intestinal contents and habitat of carp could effectively inhibit the outbreak of koi herpesvirus (KHV) (Yoshida et al., 2013). Dietary supplementation with *Bacillus* spp. reduces the mortality of Tilapia (*Oreochromis mossambicus*) infected with Tilapia lake virus (TiLV) and the levels of TiLV in various immune organs (Waiyamitra et al., 2020). An extract of *Chromobacterium aquaticum* from lake water not only improves the immunity of zebrafish (*Danio rerio*) but also has a broad-spectrum bactericidal effect on its metabolites (Yi et al., 2019). The *Serratia marcescens MS01* isolated from the intestine of largemouth bass (*Micropterus salmoides*) significantly inhibited the proliferation of *Micropterus salmoides* rhabdovirus (MSRV) (Song et al., 2023). These reports indicate that some probiotics and their metabolites in the gut can possess antiviral properties. However, changes in gut microbes and their metabolism after infection with IHNV have not been reported for rainbow trout at different culture temperatures.

With the development of high-throughput sequencing technology, combined analysis strategy of multi-omics is widely used. The metabolome reflects the activities of life that are ongoing over a period of time and the effects of environmental, physiological and pathological changes on the organism (Liu et al., 2022). Therefore, it is important to analyze changes in metabolite levels and establish the relationship between metabolites and pathogen invasion to help identify disease biomarkers and elucidate disease pathogenesis (Olive and Sassetti, 2016). Metagenomic enables the direct sequencing of microbial communities on a large scale, revealing the genetic composition of microbes in the intestine and the function of their communities (Jiang et al., 2022). In contrast to traditional microbial culture and other techniques, metagenomic have the advantages of more accurate species classification, higher sensitivity, preservation of the original proportion of the species, a wide range of species to be detected, and the avoidance of non-culturable microorganisms (Shi et al., 2023). Viruses can disrupt the balance of gut microbes thereby affecting the absorption and metabolism of nutrients in the host. Therefore, understanding host-pathogen interactions is important for IHN control.

In this study, we analyzed the changes in gut microorganisms and metabolites in rainbow trout infected with IHNV at temperatures of 12–13°C and 16–17°C using metagenomic and metabolomic analyses. To comprehend the evolving patterns of gut microorganisms and metabolites in rainbow trout infected with IHNV at various incubation temperatures, and to identify potential flora that may have preventive and curative effects on IHN. The results of this study can help to understand the immune response mechanism of rainbow trout gut microorganisms and metabolites to IHNV at different temperatures, and to explore the probiotics in rainbow trout gut that may have a preventive effect against IHN, which can provide theoretical support and new methods for the prevention and treatment of IHN.

## 2. Materials and methods

### 2.1. Statement of ethics

Experiments involving rainbow trout were carried out in accordance with the Chinese legislation on the use and care of laboratory animals. All experimental procedures involving animals were approved by the Animal Care Committee of Gansu Agricultural University.

### 2.2. Fish and experimental design

We obtained 40 healthy rainbow trout (juvenile fish with a consistent genetic background) an average weight of 238.34 ± 20.38 g without history of injury or infection from a rainbow trout farm in Gansu, China. These rainbow trout were randomly assigned into four groups (10 fish/tanks). The breeding was conducted in 3000 L tanks with oxygenated and UV-sterilized groundwater, and the water was continuously aerated. The water temperature was 12–13°C for two groups and 16–17°C for the other two groups. The fish were fed commercial trout feed daily at a feeding rate calculated to be 2% of their body weight. Residual feed and feces were cleaned up 2 h after feeding, and the water was changed twice daily, with 1/3 of the water replaced each time. Each group of fish was acclimated to a set temperature for 1 week.

**Figure 1:**
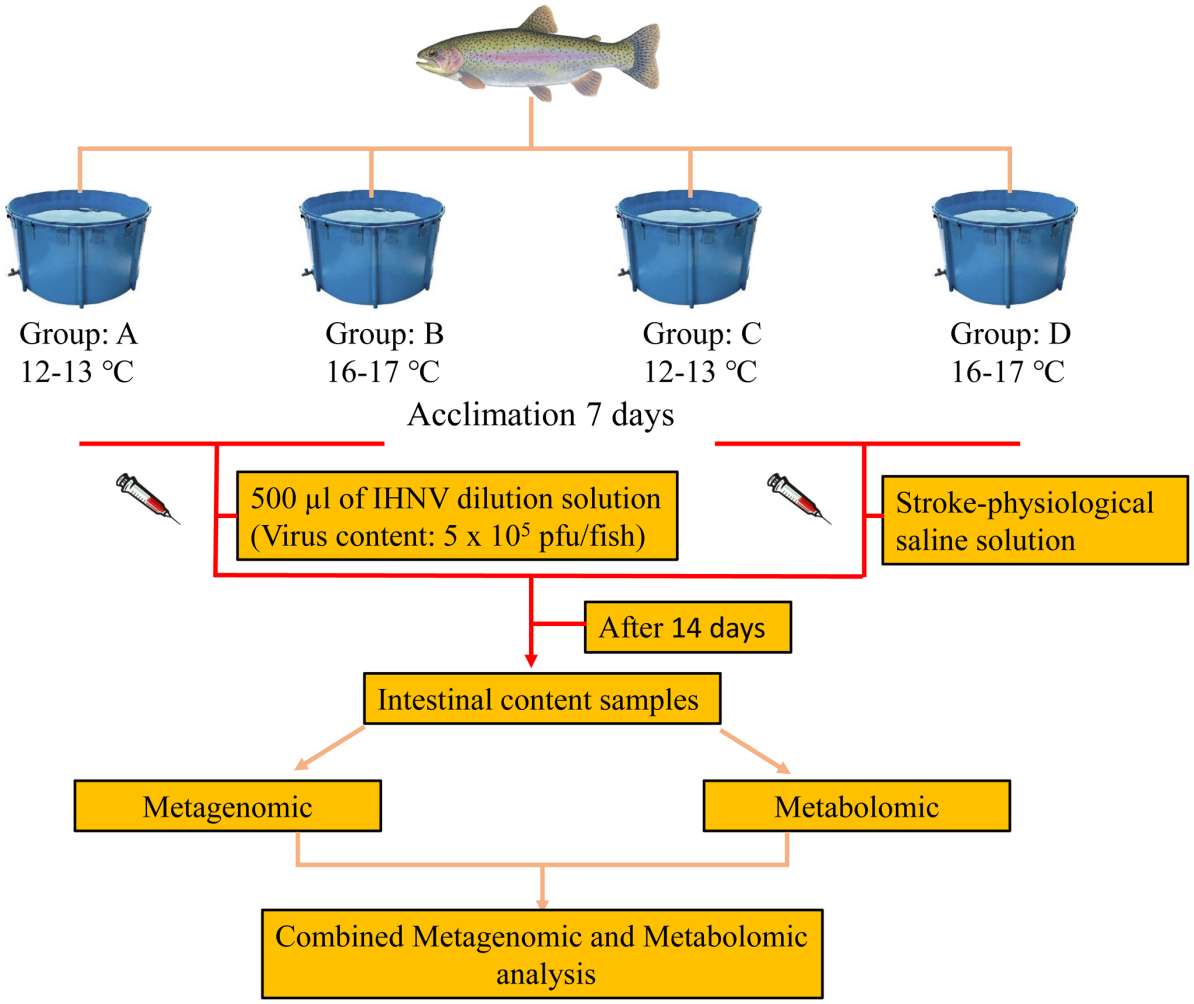
General workflow and summary of the present study.

At the end of the acclimation period, the control group was injected with 500 μL of saline in the peritoneal cavity and the treatment group was injected with 500 μL of virus mixture (Viral content: 5 × 10^5^ plaque forming units (pfu)/fish; Infectious hematopoietic necrosis virus (IHNV) strain was isolated and preserved from the diseased rainbow trout by the experimental group). The 12–13°C and 16–17°C attack groups were recorded as groups A and B, and the 12–13°C and 16–17°C control groups were recorded as groups C and D. The fish were then raised using the same management practices as during the acclimation period for a further 14 days. A schematic representation of the experimental procedure is provided in Figure 1.

After the feeding period, fish were anaesthetized with the administration of a lethal does (80.0 mg/L) of MS-222 (Sigma Aldrich Co., St. Louis, MO, United States), wiped with 75% alcohol cotton balls, and the midgut tube was removed under aseptic conditions. The contents were squeezed out and placed in a 2 mL lyophilization tube, and the inside of the tube was rinsed with PBS and the rinsing solution was thoroughly mixed with the intestinal contents. The frozen tubes were taken back to the laboratory in liquid nitrogen and saved in a −80°C refrigerator and sent on dry ice to Biomarker Technologies for sequencing.

### 2.3. Metagenome sequencing and bioinformatics analysis

We selected a total of 12 samples of intestinal contents (3 samples/group) for microbial DNA extraction. Microbial DNA was extracted from the intestinal using Bacterial DNA Extraction Kit (Omega, Shanghai, China). DNA concentration was measured using a Nanodrop 1000 (Thermo Fisher Scientific, Wilmington, DE, United States) and DNA integrity was evaluated on a 1% agarose gel. Microorganisms DNA libraries were constructed using the TruSeq DNA sample preparation kit (Illumina, San Diego, CA, United States) and sequenced by paired-end chemistry (PE150) on the Illumina Hiseq X Ten platform (Illumina, San Diego, CA, United States) from Biomarker Technologies (Beijing, China). The raw reads from sequencing were quality controlled and filtered and compared with the host genome using Bowtie2 to remove host contamination to obtain clean reads, which were assembled using MEGAHIT for macro-genome assembly and evaluated using QUAST. MetaGeneMark (Version3.26) was used for gene prediction. Construction of non-redundant gene sets using MMseqs2. The predicted sequences were compared with GenBank’s Nonredundant database (Nonredundant database, NR) by BLAST, and sequences with *E*-value ≤ 1e^−5^ were considered as meaningful and species annotation information was obtained. Finally, the predicted genes were compared with GO, KEGG, eggNOG, Pfam, SwissProt, CAZy, VFDB, CYPED and other databases to obtain gene function annotations.

### 2.4. Metabolome sequencing and bioinformatics analysis

A total of 24 samples of intestinal contents (6 samples/group, three samples are the same as the metagenome sequencing samples) were selected for metabolome sequencing. Weigh 50 mg of sample, add 1,000 μL of extract containing the internal standard (methanol: acetonitrile volume ratio = 1:1, internal standard concentration: 20 mg/L), add steel beads, grind at 45 Hz for 10 min, sonicate for 10 min (ice water bath), leave for 1 h at −20°C, then centrifuge at 4°C at 12,000 rpm for 15 min and carefully remove 500 μL of supernatant in an EP tube; The extract was dried in a vacuum concentrator, 160 μL of extract solution (acetonitrile to water volume ratio: 1:1) was added to the dried metabolites, vortexed for 30 s, sonicated in an ice-water bath for 10 min, and the samples were centrifuged at 4°C for 15 min at 12,000 rpm. The supernatant [120 μL as carefully placed in a 2 mL injection vial, and 10 μL of each sample was mixed into a quality control (QC) sample for testing on the machine]. LC–MS/MS was performed using a UHPLC system (Waters Acquity I-Class PLUS) with a UPLC HSS T3 column (1.8 μm, 2.1 × 100 mm, Waters) coupled to a Waters Xevo G2-XS QTof. The mobile phase comprising 0.1% formic acid aqueous solution (A) and 0.1% formic acid acetonitrile (B). The injection volume was 1 μL. The primary and secondary mass spectrometry data were acquired on a Waters Xevo G2-XS QTof high-resolution mass spectrometer using the MSe mode in the acquisition software (MassLynx V4.2, Waters). The raw data collected were processed by Progenesis QI software for peak extraction, peak alignment and other data processing operations, and identified based on the Progenesis QI software online METLIN database and BioMarker’s own library. Simultaneous theoretical fragment identification, within 100 ppm deviation in parent ion mass number and 50 ppm deviation in fragment ion mass number. Principal component analysis (PCA), partial least squares discriminant analysis (PLS-DA) and orthogonal partial least squares discriminant analysis of difference groupings (OPLS-DA) were applied to analyze significant metabolites between groups using the R package model. The variable importance in the projection (VIP) value of the model was calculated using multiple cross-validation. The difference multiples, *p*-values, and VIP values of the OPLS-DA model were combined to screen for differential metabolites. The screening criteria were: FC > 1, *p*-value < 0.05, and VIP > 1. Differential metabolites and associated pathways were further validated and analyzed using web-based databases, including the Human Metabolome Database (HMDB) and the Kyoto Encyclopedia of Genes and Genomes (KEGG) database.

### 2.5. Statistical analysis

All data are presented as the mean value with the standard deviation (mean ± SD) and were analyzed using independent samples *t*-test. *p*-values < 0.05 were considered statistically significant. Statistical analyses were performed with SPSS 24.0 (IBM Corp, Armonk, NY, United States).

## 3. Results

### 3.1. Macrogenome

#### 3.1.1. Macrogenome sequencing information

Illumina Hiseq sequencing of 12 rainbow trout midgut contents samples, and after quality control, removal of host contamination and data assembly yielded a total of 2,307,835 Contigs, with an average of 192,319.6/sample. The N50 averaged 478. All samples displayed a rarefaction curve that flattened out, indicating that the sequencing depth was sufficient to capture the diversity of species and functions present in these colony samples (Supplementary Figure S1).

#### 3.1.2. Differences in gut microbial groupings

**Figure 2:**
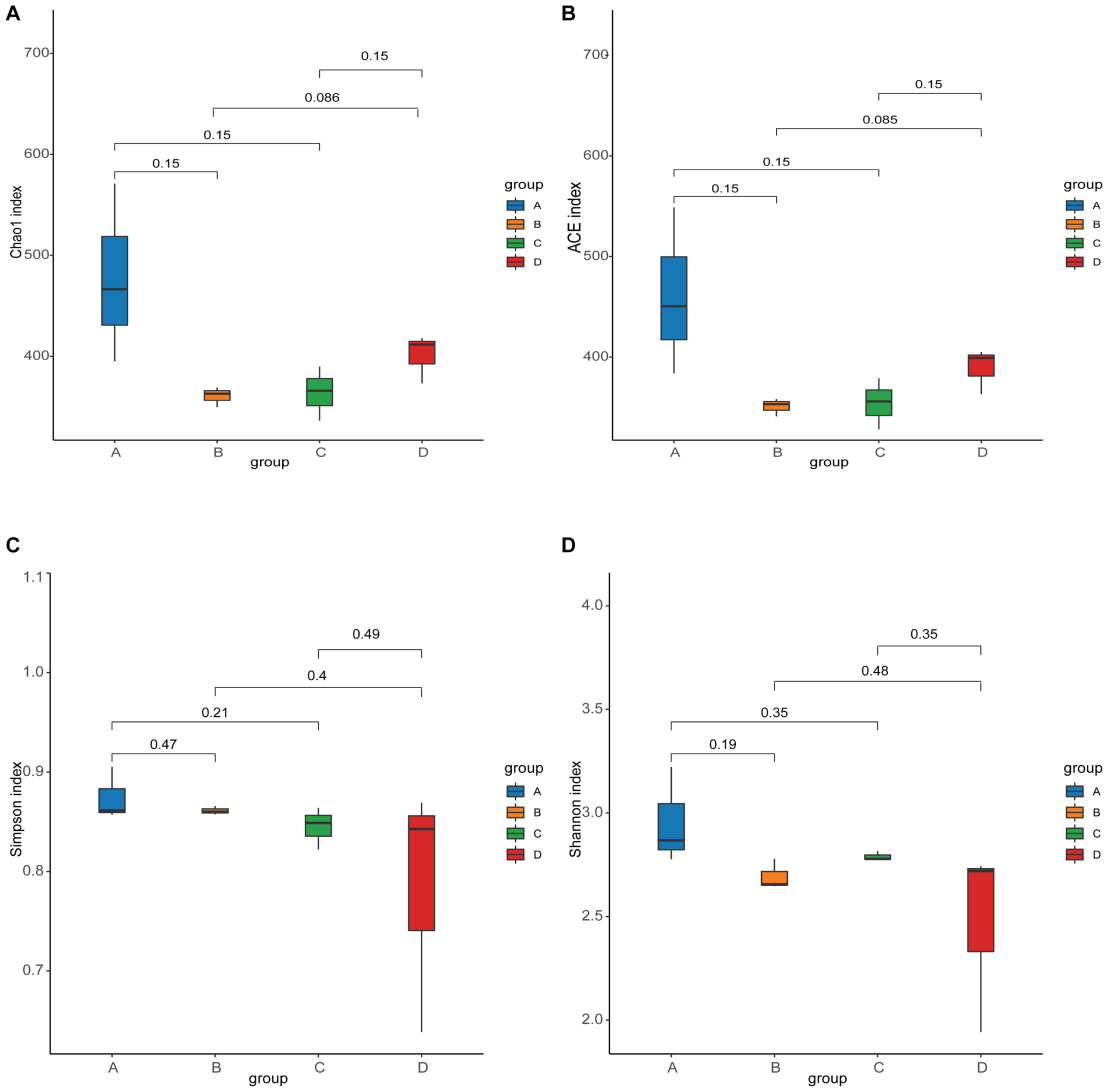
Alpha diversity of gut microbes in each group. **(A)** Chao1 index. **(B)** ACE index. **(C)** Simpson index. **(D)** Shannon index.

**Figure 3:**
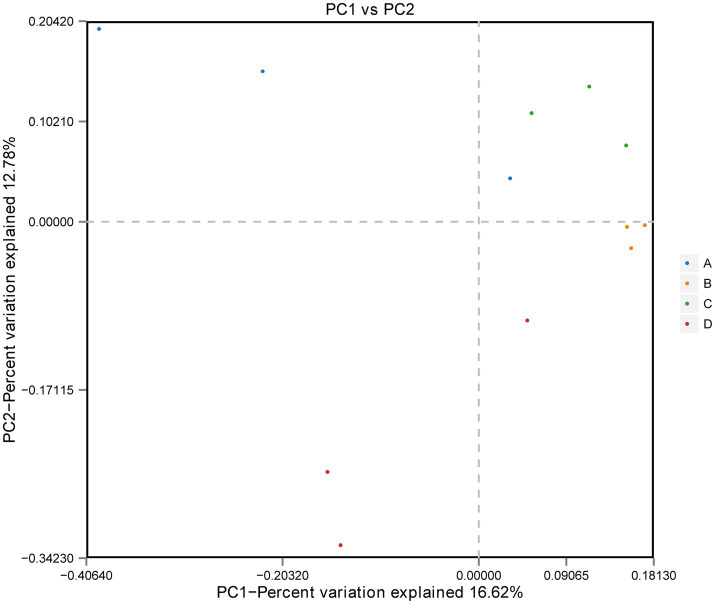
Beta diversity: principal coordinate analysis (PCoA) of bacterial community structure based on Bray-Curtis distances.

ACE and Chao1 are species richness indices, and Shannon and Simpson are species richness and evenness indices. In this study, the alpha diversity of gut microorganisms under different treatments was measured using Chao1, ACE, Simpson and Shannon indices. The differences in Chao1, ACE, Simpson and Shannon between groups A, B, C, and D were not significant (*p* > 0.05), but Chao1 and ACE in group D were higher than in group B and tended to be significant (0.05 < *p* < 0.1) (Figures 2A–D). The results show that there is a trend toward higher species richness and evenness in Group D than in Group B. The beta diversity of microbial communities was calculated and visualized by Principal Coordinates Analysis (PCoA) using Bray-Curtis distances. The results showed that the gut microbial communities of rainbow trout differed between treatment groups (Figure 3).

**Figure 4:**
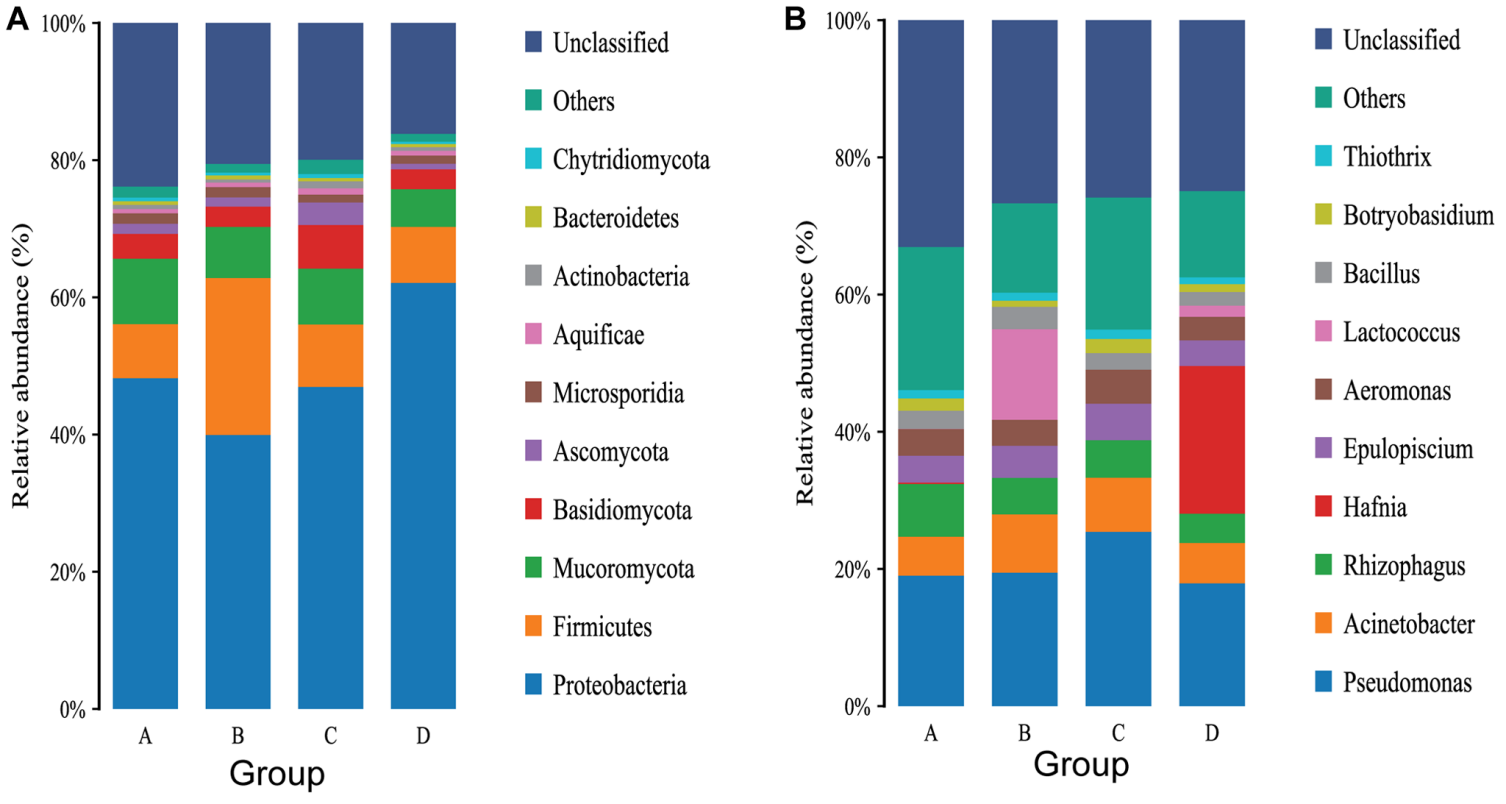
**(A)** Top 10 gut flora in terms of relative abundance of phylum levels. **(B)** Top 10 gut flora in terms of relative abundance of phylum levels.

Removing the Unassigned detected in the samples, the gut microbiota of rainbow trout was composed of Bacteria, Fungi, Viruses, Archaea, Metazoa and Eukaryota, However, Eukaryota was not found in group A (Supplementary Figure S2). At the phylum level, a total of 74 phyla were annotated in the 12 samples, with 58 phyla annotated in group A, 49 phyla in group B, 48 phyla in group C, and 51 phyla in group D (Supplementary Figure S3A). A total of 51 core phyla were identified as being common to all four groups (Supplementary Figure S3A). The dominant phyla in the rainbow trout intestinal flora are Proteobacteria, Firmicutes, Mucoromycota and Basidiomycota (Figure 4A). While there were no significant differences between Groups C and A, as well as between Groups C and D at the phylum level (Supplementary Tables S1, S4). Groups D and B significant difference was actinobacteria (D: 0.083188%, B: 0.065120%) (*p* < 0.05) (Supplementary Table S2). Groups A and B significant difference were Candidatus Parcubacteria (A: 0.000243%, B: 0.0%), Firmicutes (A: 1.078702%, B: 3.371615%) and Fusobacteria (A: 0.000132%, B: 0.001808%) (Supplementary Table S4).

At the genus level, a total of 1,005 genera were annotated in the 12 samples, with 752 genera in group A, 539 genera in group B, 561 genera in group C and 613 genera in group D (Supplementary Figure S3B). There were 343 core genera common to all four groups (Supplementary Figure S3B). The dominant genus in group A were *Pseudomona*, *Rhizophagus* and *Acinetobacter* (Figure 4B). The dominant genera in group B were *Pseudomonas*, *Lactococcus* and *Acinetobacter* (Figure 4B). The dominant genera in group C were *Pseudomonas*, *Acinetobacter* and *Rhizophagus* (Figure 4B). The dominant genera in group D were *Hafnia*, *Pseudomonas* and *Acinetobacter* (Figure 4B). Infection with IHNV can induce changes in the gut microbiota of rainbow trout, and there are differences in the genus infected with IHNV under different aquaculture temperatures. There are 23 significantly different genera between group C and group A, with 24 genera being significantly different between group D and group B, 29 genera being significantly different between group A and group B, and 33 genera being significantly different between group C and group D.

**Figure 5:**
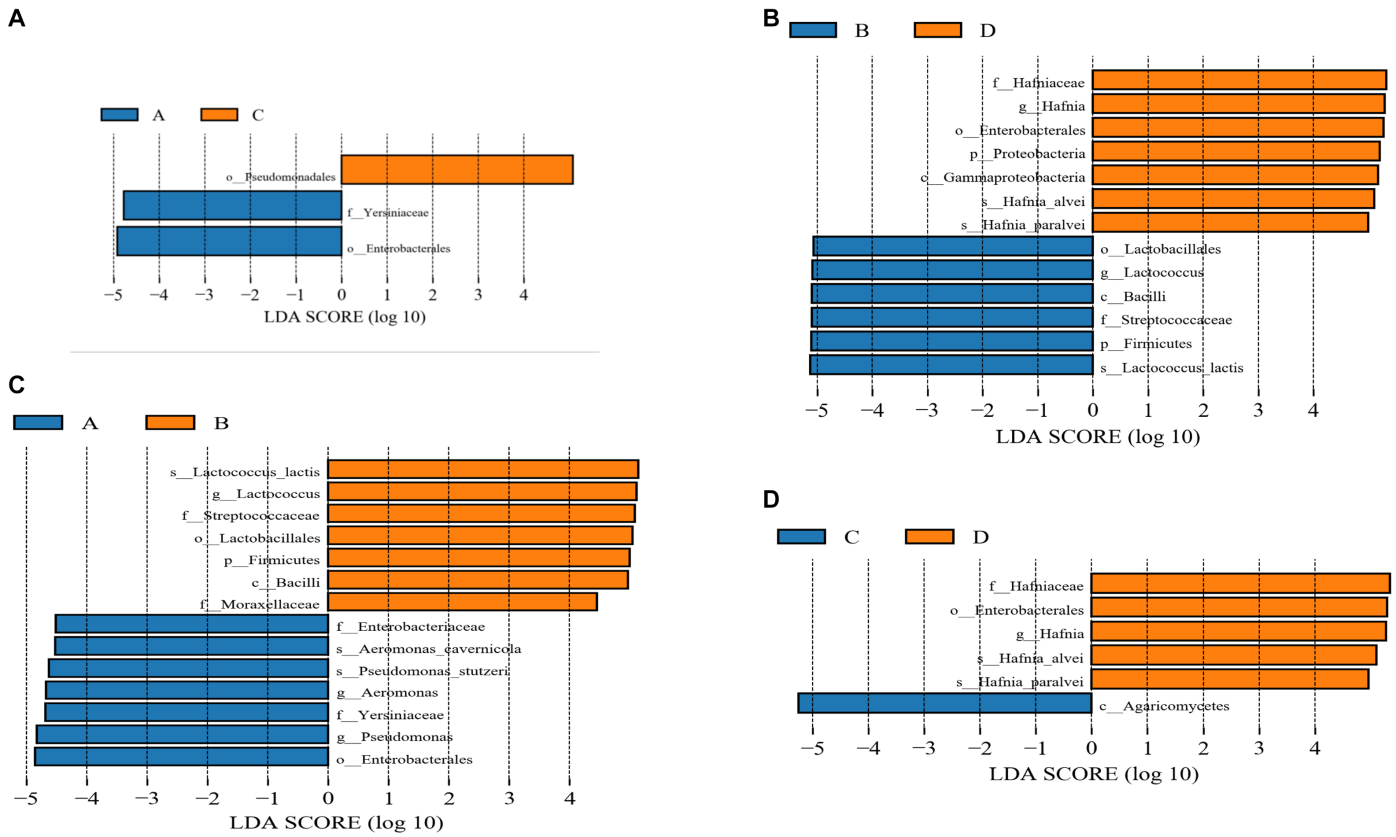
**(A)** Linear discrimination analysis (LDA) effect size (LEfSe) analysis comparing C and A groups. **(B)** Linear discrimination analysis (LDA) effect size (LEfSe) analysis comparing D and B groups. **(C)** Linear discrimination analysis (LDA) effect size (LEfSe) analysis comparing A and B groups. **(D)** Linear discrimination analysis (LDA) effect size (LEfSe) analysis comparing C and D groups.

To evaluate further differences in the microbial communities of the gut contents of the four groups of rainbow trout and to screen for specific biomarkers, Linear discriminant analysis Effect Size (LEfSe) (LDA > 4) was used. The results indicated that the dominant microbiota of group C and group A differed. The dominant flora in group C was Pseudomonadales, while in group A it consisted of Yersiniaceae and Enterobacterales (Figure 5A). Compared with group D, the dominant flora in group B were Lactobacillales, *Lactococcus*, Bacilli, Streptococcaceae, Firmicutes and *Lactococcus lactis*. On the other hand, the dominant flora in group D consisted of Hafniaceae, *Hafnia*, Enterobacterales, Proteobacteria, Gammaproteobacteria, *Hafnia alvei* and *Hafnia paralvei* (Figure 5B). The dominant microbial communities differ between Group A and Group B. The dominant groups in Group A were Enterobacteriaceae, *Aeromonas cavernicola*, *Pseudomonas stutzeri*, *Aeromonas*, Yersiniaceae, *Pseudomonas* and *Enterobacterales*, and the dominant flora in Group B were *Lactococcu lactis*, *Lactococcus*, Streptococcaceae, Lactobacillales, Firmicutes, Bacilli and Moraxellaceae (Figure 5C). Compared to group D, the dominant flora in group C were Agaricomycetes, while Hafniaceae, Enterobacterales, *Hafnia*, *Hafnia alvei*, and *Hafnia paralvei* were the dominant flora in group D (Figure 5D).

#### 3.1.3. Functional analysis of intestinal microbes

At the gene level, a total of 58,827 non-redundant genesets were detected and were annotated to 5,943 KOs. The total length is 28,525,935 bp with an average length of 484.00 bp. The Max length is13,434 bp and the Min length 102 bp (Table 1).

**Table 1:** Non-redundant Geneset set result statistics.

Type	Geneset number	Total length (bp)	Average (bp)	Max length (bp)	Min length (bp)
Geneset	58,827	28,525,935	484.0	13,434	102

**Figure 6:**
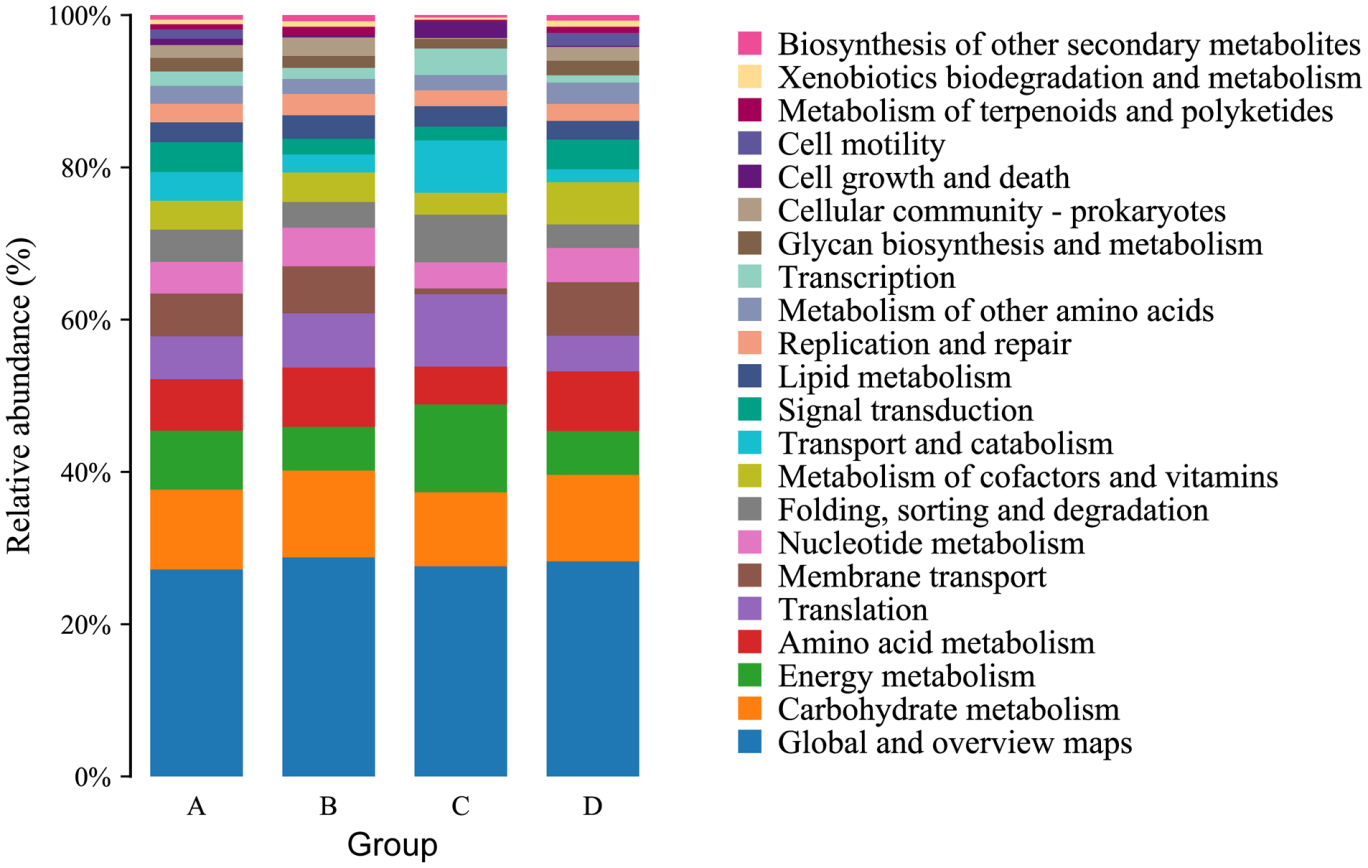
Functional notes on various groups of microorganisms at KEGG-pathway level 2.

**Figure 7:**
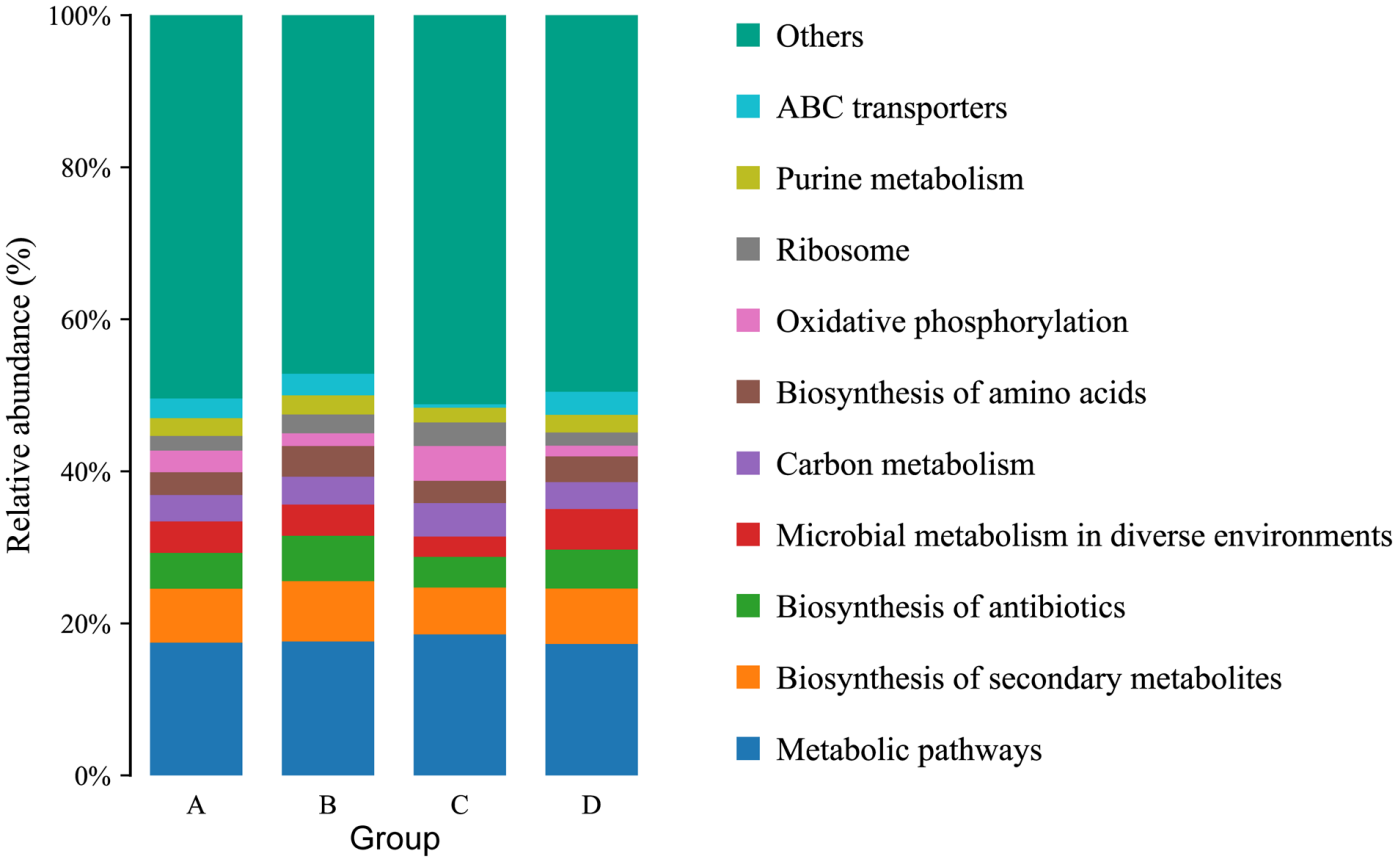
Functional notes on various groups of microorganisms at KEGG-pathway level 3.

The function of the intestinal microbiome was determined by KEGG map. For KEGG analysis, a total of 22 pathways were annotated at the second level. The top four pathways with the most enriched number of annotated genes in group A were Global and overview maps, Carbohydrate metabolism, Energy metabolism and Amino acid metabolism, in groups B and C were Global and overview maps, Carbohydrate metabolism, Amino acid metabolism and Translation, and in group D were Global and overview maps, Carbohydrate metabolism, Amino acid metabolism and Membrane transport (Figure 6). At the third level, 157 pathways were observed. The top three pathways with the most enriched number of annotated genes in groups A and B were Metabolic pathways, Biosynthesis of secondary metabolites and Biosynthesis of antibiotics, in group C were Metabolic pathways, Biosynthesis of secondary metabolites and Oxidative phosphorylation and in group D were Metabolic pathways, Biosynthesis of secondary metabolites and Microbial metabolism in diverse environments (Figure 7). The differential pathways between groups C and A were Carbon fixation in photosynthetic organisms (C: 0.009683%, A: 0.005666%), Fatty acid elongation (C: 0.000886%, A: 0.000355%), Photosynthesis (C: 0.012486%, A: 0.006821%) and Glycosphingolipid biosynthesis-ganglio series (C: 0.000404%, A: 0%), the differential pathways between groups D and B were Other types of O- glycan biosynthesis (D: 0.000277%, B: 0.000647%), the different pathways between groups A and B were Ubiquitin mediated proteolysis (A: 0.007503%, B: 0.005117%), the different pathways between groups C and D were Photosynthesis (C: 0.012486%, D: 0.007512%).

### 3.2. Metabolic

#### 3.2.1. Differential metabolites of intestinal contents under different treatments

**Figure 8:**
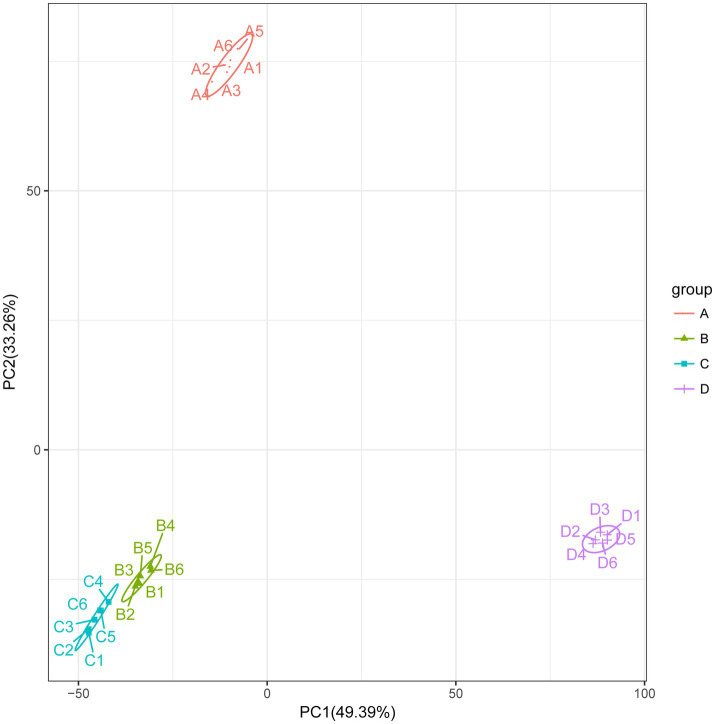
Principal coordinate analysis (PCoA) of intestinal metabolites.

To better understand the changes in metabolites in different treatments, based on the LC-QTOF platform, the metabolome was analyzed qualitatively and quantitatively in 24 samples, and a total of 5,847 metabolites were detected in both positive and negative modes (2,743 metabolites in positive mode and 3,104 in negative mode). The PCA results for clustering showed a marked separation between the groups, which indicated that the metabolites in the samples changed significantly after the different treatments (Figure 8).

**Figure 9:**
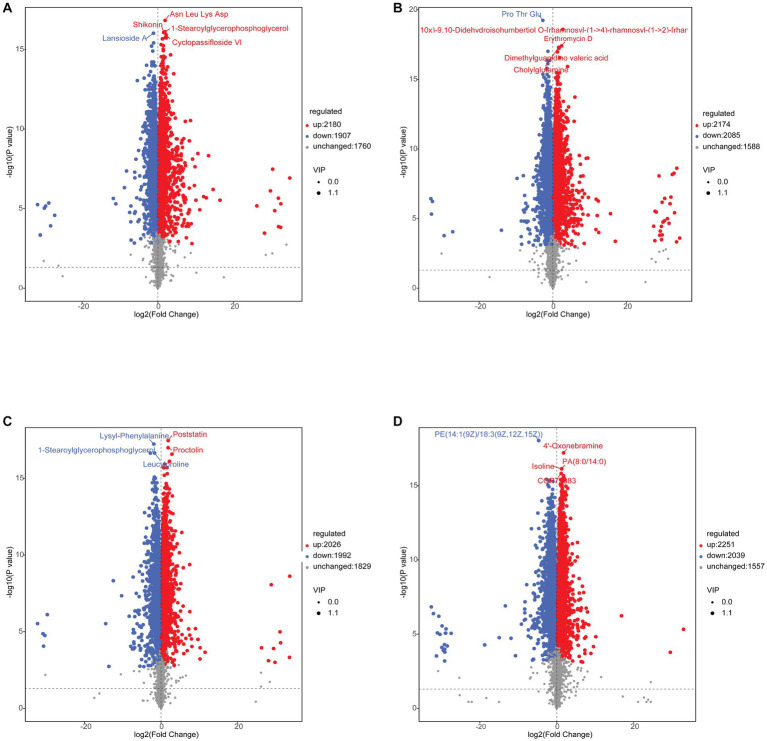
**(A)** C vs. A differential metabolite Volcano Plot. **(B)** C vs. A differential metabolite Volcano Plot. **(C)** C vs. A differential metabolite Volcano Plot. **(D)** C vs. A differential metabolite Volcano Plot.

After screening the relative concentrations of intestinal metabolites by VIP (VIP > 1), *t*-test (*p* < 0.05) and FC (FC > 1 or < 0.5). The results showed a total of 4,087 (up-regulated: 2,180, down-regulated: 1,907) differential metabolites for C vs. A (Figure 9A), 4,259 (up-regulated: 2,174, down-regulated: 2,085) differential metabolites for D vs. B (Figure 9B), 4,018 (up-regulated: 2,026, down-regulated: 1,992) differential metabolites for the A vs. B group (Figure 9C), and 4,290 (up-regulated: 2,251, down-regulated: 2,039) differential metabolites for the C vs. D group (Figure 9D). To explore further the metabolites associated with organismal immunity following infection with IHNV at different culture temperatures, we selected metabolites associated with immune, Cell growth and death and among others (Table 2), to explore their important functions in the host.

**Table 2:** Differential metabolites of the intestinal contents of rainbow trout in different treatments.

Metabolites	C vs. A				D vs. B				A vs. B							C vs. D
	Log_2_FC	*p*	VIP	Changes	Log_2_FC	*p*	VIP	Changes	Log_2_FC	*p*	VIP	Changes	Log_2_FC	*p*	VIP	Changes
3′,5′-Cyclic AMP	−1.26	0.00	1.10	Down	−0.56	0.00	1.05	Down	0.69	0.00	1.07	Up	0.00	0.99	0.00	Unchanged
Ecdysone	0.85	0.00	1.03	Up	0.39	0.07	0.60	Unchanged	−0.36	0.04	0.70	Unchanged	0.10	0.56	0.20	Unchanged
1-Octadecanoyl-sn-glycero-3-phosphoethanolamine	1.43	0.00	1.12	Up	0.41	0.00	1.09	Up	−1.36	0.00	1.14	Down	−0.34	0.00	1.08	Down
L-Glutamate	0.47	0.00	1.11	Up	0.51	0.00	1.10	Up	−0.51	0.00	1.13	Down	−0.54	0.00	1.08	Down
Glutathione	7.30	0.00	1.12	Up	−3.56	0.00	1.10	Down	−3.22	0.00	1.13	Down	7.64	0.00	1.09	Up
Fibrin	−0.74	0.00	1.12	Down	1.66	0.00	1.08	Up	0.37	0.00	0.95	Unchanged	−2.04	0.00	1.09	Down
Prostaglandin H2	−0.58	0.00	1.12	Down	1.33	0.00	1.11	Up	0.22	0.00	1.12	Up	−1.69	0.00	1.09	Down
Leukotriene C4	1.17	0.00	1.10	Up	−0.23	0.02	0.75	Unchanged	−1.02	0.00	1.10	Down	0.38	0.00	0.96	Unchanged
Leukotriene D4	0.67	0.00	1.06	Up	−0.92	0.00	1.08	Down	−0.50	0.00	1.00	Unchanged	1.09	0.00	1.08	Up
Angiotensin II	1.53	0.00	1.12	Up	0.42	0.00	0.91	Unchanged	−1.03	0.00	1.13	Down	0.08	0.46	0.27	Unchanged
Angiotensin (1–7)	−1.40	0.00	1.12	Down	−0.34	0.00	0.97	Unchanged	0.40	0.00	1.06	Up	−0.66	0.00	1.06	Down
Sphingosine	−0.23	0.10	0.57	Unchanged	1.33	0.00	1.02	Up	0.16	0.26	0.41	Unchanged	−1.40	0.00	1.02	Down
1-Octadecanoyl-2-(15S-hydroxy-5Z,8Z,11Z,13E-eicosatetraenoyl)-sn-glycero-3-phosphoethanolamine	−0.39	0.00	0.96	Unchanged	4.43	0.00	1.10	Up	0.17	0.08	0.61	Unchanged	−4.65	0.00	1.08	Down
ADP	0.69	0.00	0.95	Unchanged	−0.80	0.00	1.04	Down	−0.54	0.00	0.93	Unchanged	0.95	0.00	1.03	Up
cGMP	−0.12	0.28	0.39	Unchanged	−2.11	0.00	1.10	Down	0.22	0.07	0.63	Unchanged	2.21	0.00	1.08	Up
Retinoate	−0.19	0.01	0.78	Unchanged	0.69	0.00	1.03	Up	−0.17	0.03	0.72	Unchanged	−1.06	0.00	1.06	Down
Sphingosine 1-phosphate	−0.09	0.00	0.99	Unchanged	−0.17	0.00	1.06	Down	−0.54	0.00	1.13	Down	−0.46	0.00	1.09	Down
gamma-L-Glutamyl-L-cysteine	−1.03	0.00	0.89	Unchanged	−0.21	0.25	0.41	Unchanged	2.49	0.00	1.11	Up	1.67	0.00	0.99	Unchanged

#### 3.2.2. Key differential activation pathways in rainbow trout under different treatments

**Figure 10:**
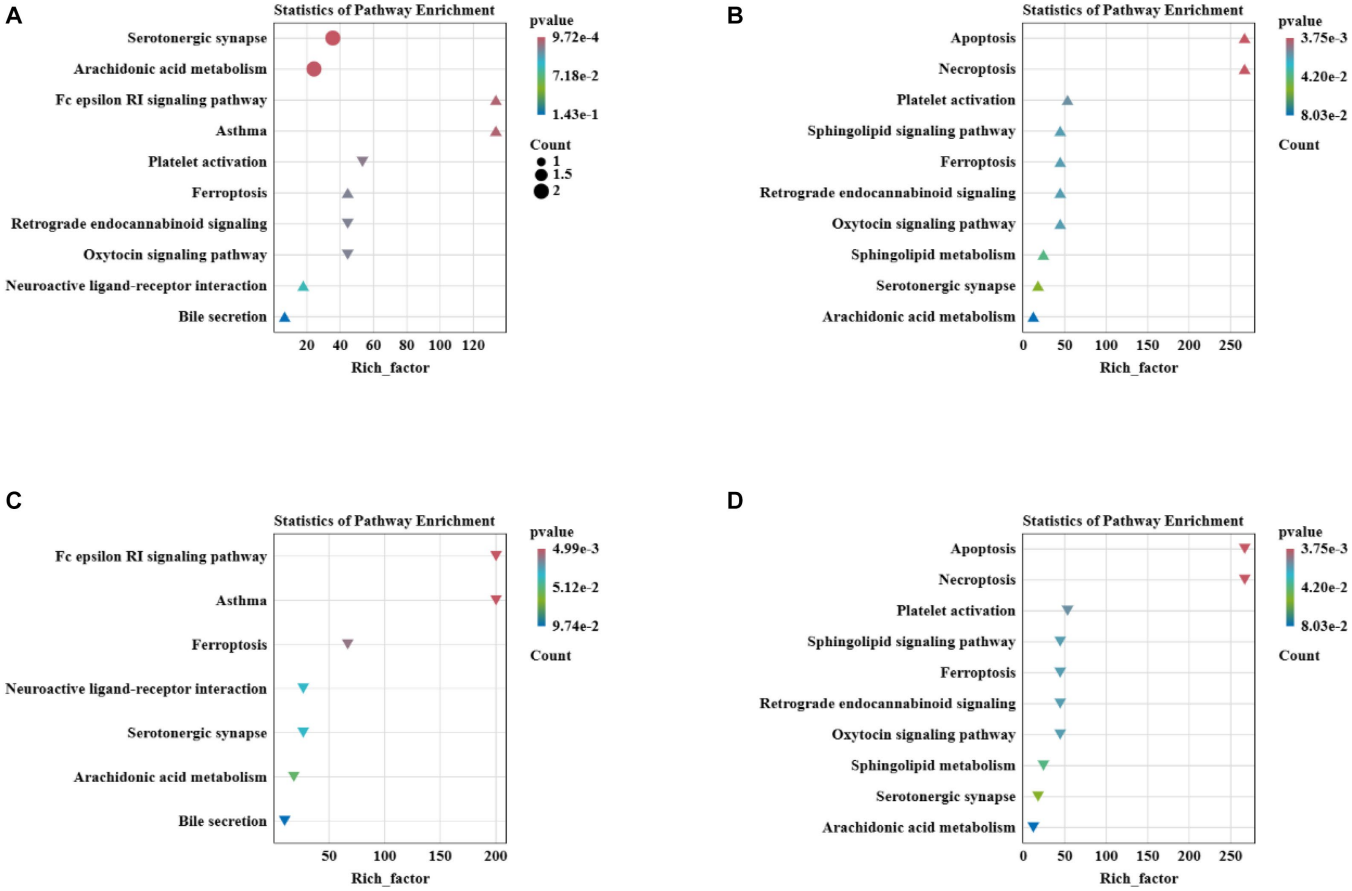
**(A)** Metabolic pathway enrichment analysis for C vs. A differential metabolites. **(B)** Metabolic pathway enrichment analysis for D vs. B differential metabolites. **(C)** Metabolic pathway enrichment analysis for A vs. B differential metabolites. **(D)** Metabolic pathway enrichment analysis for C vs. D differential metabolites.

To further explore the activation of potential metabolic pathways in rainbow trout by IHNV at different farming temperatures, we used the KEGG map to correlate these important metabolites and metabolic pathways. The differential metabolites in the C vs. A group were enriched in Serotonergic synapse, Arachidonic acid metabolis, Platelet activation, Retrograde endocannabinoid signaling, Oxytocin signaling pathway, Fc epsilon RI signaling pathway, Asthma, Ferroptosis, Neuroactive ligand-receptor interaction and Bile secretion pathway (Figure 10A). D vs. B differential metabolites were mainly enriched in Apoptosis, Necroptosis, Platelet activation, Sphingolipid signaling pathway, Ferroptosis, Retrograde endocannabinoid signaling, Oxytocin signaling pathway, Sphingolipid metabolism, Serotonergic synapse and Arachidonic acid metabolism (Figure 10B). Differential metabolites in A vs. B were enriched in the Fc epsilon RI signaling pathway, Asthma, Ferroptosis, Neuroactive ligand-receptor interaction, Serotonergic synapse, Arachidonic acid metabolism and Bile secretion (Figure 10C). The main metabolites of C vs. D are enriched in Apoptosis, Necroptosis, Platelet activation, Sphingolipid signaling pathway, Ferroptosis, Retrograde endocannabinoid signaling, Oxytocin signaling pathway, Serotonergic synapse and Arachidonic acid metabolism, Oxytocin signaling pathway, Sphingolipid metabolism, Serotonergic synapse and Arachidonic acid metabolism (Figure 10D).

### 3.3. Correlation analysis of microorganisms, metabolites

**Figure 11:**
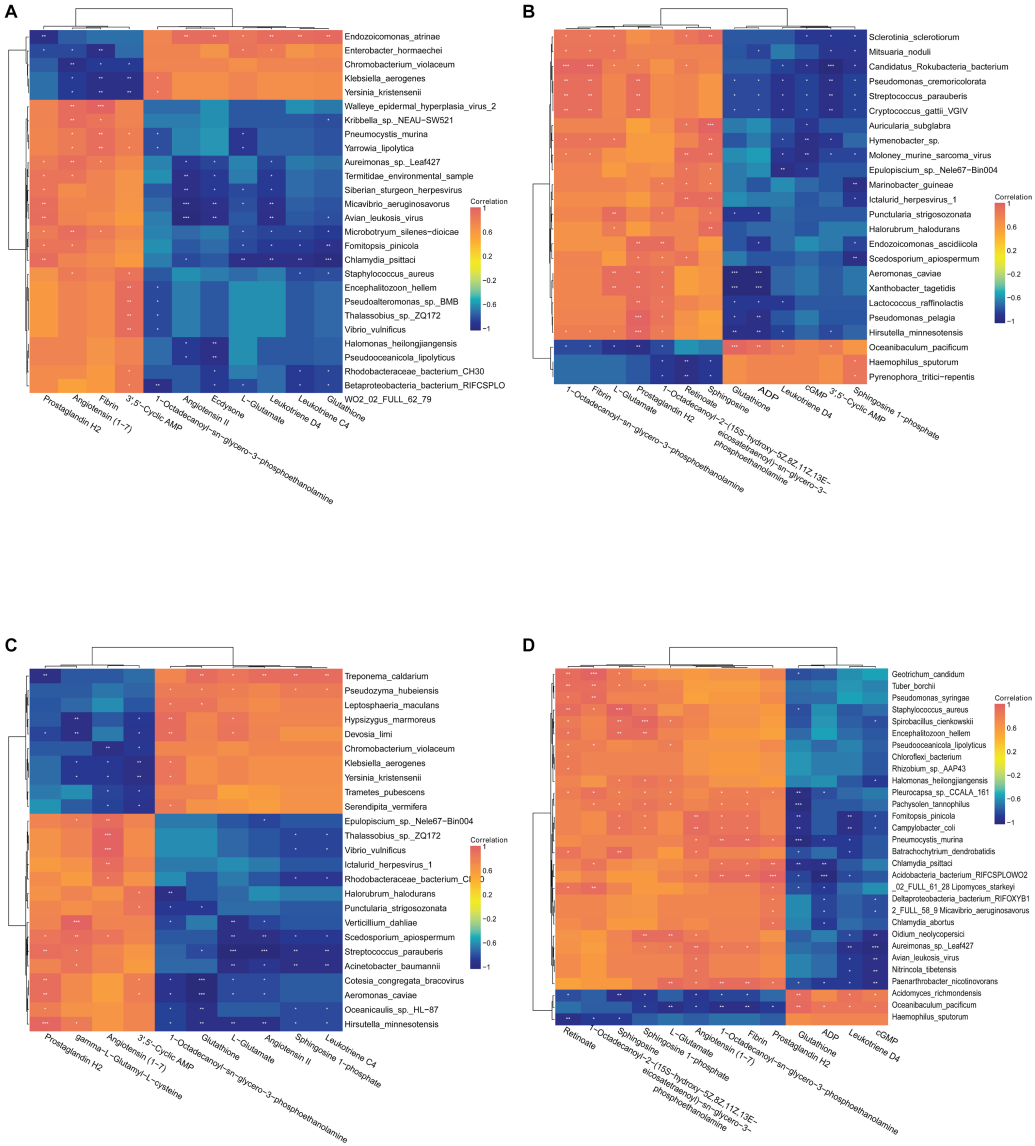
**(A)** Correlation analysis of C vs. A differential metabolites with species abundance. **(B)** Correlation analysis of D vs. B differential metabolites with species abundance. **(C)** Correlation analysis of A vs. B differential metabolites with species abundance. **(D)** Correlation analysis of C vs. D differential metabolites with species abundance.

To understand the relationship between important differential metabolites and microorganisms, Pearson correlation analysis was performed. In the C vs. A group, the metabolites of concern were found to be correlated with 26 microorganisms (Figure 11A). *Staphylococcus aureus* was positively correlated with the up-regulated metabolites Leukotriene C4 and Glutathione, and significantly negatively correlated with the down-regulated metabolites 3′,5′-Cyclic AMP and Angiotensin (1–7). *Vibrio vulnificus* showed a negative correlation with the up-regulated metabolite 1-Octadecanoyl-sn-glycero-3-phosphoethanolamine and a positive correlation with the down-regulated metabolite 3′,5′-Cyclic AMP. *Yersinia kristensenii* was negatively associated with the up-regulated metabolite 1-Octadecanoyl-sn-glycero-3-phosphoethanolamine, and significantly positively associated with the down-regulated metabolites 3′,5′-Cyclic AMP, Fibrin and Angiotensin (1–7). *Fomitopsis pinicola* was negatively associated with the up-regulation of the metabolites Glutathione, L-Glutamate, Leukotriene C4, Leukotriene D4, as well as with the down-regulation of the metabolites Angiotensin (1–7) and Prostaglandin H2. In the D vs. B group, the differential metabolites of concern were found to be associated with 24 microorganisms (Figure 11B). *Streptococcus parauberis* was negatively correlated with the down-regulation of the metabolites Sphingosine 1-phosphate, 3′,5′-Cyclic AMP, cGMP, Leukotriene D4, ADP and Glutathione, and positively associated with the up-regulation of the metabolites Prostaglandin H2, Fibrin and 1-Octadecanoyl-sn-glycero-3-phosphoethanolamine. *Lactococcus raffinolactis* was negatively associated with the down-regulated metabolites Leukotriene D4, ADP and Glutathione, and positively associated with the up-regulated metabolites 1-Octadecanoyl-sn-glycero-3-phosphoethanolamine and Prostaglandin H2. In the A vs. B group, metabolites of focus were correlated with 25 microorganisms (Figure 11C). *Streptococcus parauberis* showed a negative correlation with the down-regulated metabolites Leukotriene C4, Sphingosine 1-phosphate, Angiotensin II, L-Glutamate and Glutathione, and positively correlated with the up-regulated metabolites gamma-L-Glutamyl-L-cysteine and Prostaglandin H2. *Hypsizygus marmoreus* showed a positive correlation with the down-regulated metabolites L-Glutamate and 1-Octadecanoyl-sn-glycero-3-phosphoethanolamine, and a negative correlation with the up-regulated metabolites 3′,5′-Cyclic AMP and gamma-L-Glutamyl-L-cysteine. In C vs. D, metabolites of concern were correlated with 30 microorganisms (Figure 11D). *Staphylococcus aureus* was negatively correlated with the up-regulated metabolite Glutathione, and positively correlated with the down-regulated metabolites Sphingosine 1-phosphate, Sphingosine and 1-Octadecanoyl-2-(15S-hydroxy-5Z,8Z,11Z,13E-eicosatetraenoyl)-sn-glycero-3-phosphoethanolamine Retinoate.

## 4. Discussion

Gut microbes, known as the “external organs” of the host, interact with and play an important role in nutrient metabolism and immune regulation (O'Hara and Shanahan, 2006; Valdes et al., 2018; Holt et al., 2021). The intestinal microflora mainly consists of Bacteria, Fungi, Viruses, Archaea, Metazoa, and Eukaryota. Under steady state conditions, the host and microorganisms interact with each other to establish a dynamic balance of mutualistic symbiosis. The host’s immune system monitors the intestinal microorganisms maintain the stability of the intestinal flora and promote the health of the intestinal mucosa, thereby enhancing the host’s resistance against pathogens (Iacob et al., 2018; Gao et al., 2020). However, different factors (food changes, antibiotic use, invasion of pathogenic bacteria, etc.) can lead to changes in the structure of the flora, disrupting the balance of the flora composition, altering the metabolic network of the flora, causing ecological imbalances, and affecting the immune-regulatory network that suppresses intestinal inflammation, thus decreasing the host’s ability to limit the invasion of the flora, and allowing pathogenic bacteria to enter the tissues to cause disease, which can ultimately lead to inflammatory bowel disease or other illnesses (Ding et al., 2017). Fish are thermotrophs, and temperature significantly affects the gut microbial communities of fish. The susceptibility of rainbow trout to IHNV is greatly influenced by temperature, leading to substantial differences in fish tolerance. Spring and fall are the peak seasons for rainbow trout IHN when the water temperature is below 15°C, while the incidence of rainbow trout IHN decreases significantly above 15°C. Based on the above analysis and findings, we hypothesize that there may be a relationship between the composition of gut microbiota in rainbow trout, their metabolites, and the increased resistance to IHN under high temperature conditions. Thus, we analyzed the composition and differences of intestinal microorganisms and their metabolites in rainbow trout infected with IHNV cultured at 12–13°C and 16–17°C using metagenomic and metabolomic analyses. We also screened for intestinal microorganisms and their metabolites that may have defensive or therapeutic effects against IHN. In this study, the intestinal microbes of rainbow trout were predominantly bacterial, which is similar to the gut composition of other fish species (Li et al., 2015). However, no Eukaryotic microorganisms were found in the intestinal microbiota following infection with IHNV at a culture temperature of 12–13°C. It has been noted that eukaryotes are less adaptable to their environment than other microorganisms (Massana and Logares, 2013), while it has been shown that the diversity of gut eukaryotes is positively correlated with host health (Lozupone et al., 2012; Rook et al., 2013). Infection of rainbow trout with IHNV under low-temperature aquaculture resulted in the depletion of eukaryotic organisms in their intestinal tract. It is further shown that infection with IHNV under low-temperature culture conditions resulted in the dysbiosis of rainbow trout intestinal microorganisms and the reduction of host innate immunity. At the phylum level, the Proteobacteria and Firmicutes were the dominant phyla in all the experimental groups, which is consistent with the results of previous studies (Huang et al., 2022). Actinomycetes, the major bacteria, produce metabolites that have ant and antibacterial activities (Valliappan et al., 2014; Landwehr et al., 2016). A significant decrease in the abundance of actinomycete was detected on days 4 and 14 after infection with IHNV (Huang et al., 2022). This further indicates that infection of rainbow trout with IHNV may cause changes in Actinobacteria. The Firmicutes can produce lactic acid, which has a regulatory function on intestinal diseases (Ganesan et al., 2018), while Fusobacteria can stimulate an inflammatory response in the host and inhibit the growth of certain pathogens (Kelly et al., 2018). A recent study has revealed that the higher antioxidant and anti-inflammatory abilities of Asian seabass (*Lates calcarifer*) are correlated with significant increases in the abundances of Firmicutes and Fusobacteria (Siddik et al., 2020). The results of this study showed that Firmicutes and Fusobacteria were significantly higher in the high-temperature attack group compared to in the low-temperature attack group. This further suggests that these two phyla are closely related to host health, and that the gut flora of the high-temperature attack group is more beneficial to host health compared to the low-temperature attack group. Different culture temperatures generally not result in significant differences in rainbow trout gut flora gate levels (Huyben et al., 2018), and similar results were found in this study.

To further understand the differences in gut flora between treatments and the impact on host health. We used LEfSE analysis to find species that differed significantly in abundance between groups and to discuss the results. The *Yersinia* within the Yersiniaceae can cause sepsis in humans and animals (Huang et al., 2023). The results of the study showed a significant increase in Yersiniaceae after infection with IHNV at 12–13°C. We hypothesized that the increase in conditionally pathogenic bacteria after infection with IHNV in rainbow trout cultured at low-temperature would further exacerbate the pathogenesis of IHN. *Lactococcus lactis*, as a probiotic, enhances the innate immune system of rainbow trout and protects the host from pathogens (Santibañez et al., 2021). Some pathogenic bacteria in the Streptococcaceae can cause various septic diseases in their host (Neila-Ibáñez et al., 2023). Significant increase in *Lactococcus lactis* and Streptococcaceae following infection with IHNV at 16–17°C. This indicates that the increase in intestinal pathogenic bacteria after IHNV infection at 16–17°C was accompanied by a significant increase in the number of some beneficial bacteria, which may contribute to resistance against IHN. Compared to group B, harmful bacteria such as *Aeromonas cavernicola*, *Pseudomonas stutzeri*, *Aeromonas*, Yersiniaceae and *Pseudomonas* were dominant in group A. On the other hand, Streptococcaceae and *Lactococcus lactis* were significantly increased in Group B. This showed that both IHNV infections led to an increase in harmful flora in the intestinal flora, but the species and abundance of harmful flora infected with IHNV at low temperatures were more high temperature.

The ability to directly perform functional analysis of microbial communities one of the most important applications of the metagenomic technique. Microbial photosynthesis and carbon fixation are similar to plant photosynthesis in that they utilize CO_2_ and H_2_O to synthesize organic matter for cell formation, while also releasing O_2_ (Robert Tabita, 2004). Microbial photosynthesis in the gut mainly originates from microalgae and photosynthetic bacteria, with microalgae being able to enhance host immunity (Cerezuela et al., 2012). Adding photosynthetic bacteria to shrimp feed can improve survival rate (Saejung et al., 2021). The carbon fixation in photosynthetic organisms and the photosynthesis functions of gut microorganisms were significantly decreased in rainbow trout infected with IHNV at 12–13°C. We hypothesized that infection with IHNV affects the abundance and function of microalgae and photosynthetic bacteria in the gut, leading to a decrease in oxygen levels within the body of rainbow trout. The occurrence of also influenced by dissolved oxygen, and we hypothesize that IHNV infection in rainbow trout at low-temperature results in a decreased ability of gut bacteria to release oxygen, thereby increasing mortality. Interestingly, the low temperature farming group photosynthesis of gut microorganisms was significantly higher than that of the high-temperature culture group, the function of photosynthesis of gut microorganisms was reduced at the optimal temperature for rainbow trout farming. It has been found that the energy metabolism of *Symphysodon aequifasciatus* increases under low-temperature stress, which is used to maintain the stability of the internal environment of the organism (Wen et al., 2017). We hypothesize that rainbow trout need to increase energy production through microbial photosynthesis to maintain the stability of the host internal environment under low-temperature culture. Meanwhile, the function of fatty acid elongation in intestinal microorganisms was significantly decreased after infection with IHNV at 12–13°C. Fatty acid elongation was found to be significantly reduced in mice with colitis (Zhu et al., 2021). This suggests that infection with IHNV during low-temperature culture may lead to inflammation in the intestine of rainbow trout. O-glycan can inhibit the effect of harmful bacteria on the host (Zhu et al., 2021; Takagi et al., 2022). The function of other types of O-glycan biosynthesis significantly decreased after infection with IHNV at 16–17°C. We hypothesize that the function of the gut microflora after infection with IHNV at high temperatures is directed toward unfavorable host health. Ubiquitin-mediated proteolysis can degrade mis-expressed proteins (Zhao et al., 2010). Ubiquitin mediated proteolysis was significantly higher in group A than in group B. We hypothesize that infection with IHNV at low temperatures has a greater impact on normal biological processes in rainbow trout, resulting in an increased burden on microbial Ubiquitin mediated proteolysis. The differences in microbial abundance and function among the different groups suggest that infection with IHNV leads to dysbiosis of gut microbiota in rainbow trout. Overall, there is an increase in harmful bacteria in the gut microbiota after infection with IHNV, which further affects the health of the host. More harmful bacteria in the gut of IHNV infected at low-temperature than at high-temperature may be due to the fact that the organism is more stable at high-temperature, and IHNV has less impact on gut and host homeostasis, and the host is more immune. Therefore, the maintenance of gut microbial homeostasis is very important in the prevention and treatments of IHN.

The intestinal microbial metabolites can enter the body’s circulation through the intestinal epithelial cells and influence various organs of the host to respond to pathogens, playing an important role in the host’s immune system (Alwin and Karst, 2021). The different treatments not only affect variety of intestinal microorganisms but also alter intestinal metabolites and metabolic pathways. For example, changes in the Serotonergic synapse pathway impact the function’s brain (Xiao et al., 2021). Arachidonic acid metabolis is significantly correlated with functions such as stress resistance and immunity in fish (Xu et al., 2022). Both the serotonergic synapse and arachidonic acid metabolism pathways are closely correlated with pain generation in the body (Hale and Lowry, 2011). The Platelet activation is not only associated with thrombosis but also affects natural and adaptive immunity and organ damage, the activated platelets produce soluble factors and interact directly with immune cells to promote inflammatory phenotypes and autoimmune responses (Zhang et al., 2022; Scherlinger et al., 2023). Retrograde endocannabinoid signaling can improve intestinal function (Zhang et al., 2020). Ferroptosis is associated with the metabolism of amino acids, lipids, and sugars as well as the development of diseases such as inflammation and cancer (Sun et al., 2020; Jiang et al., 2021). Our results showed that Serotonergic synapsevs, Arachidonic acid metabolis, Platelet activation, Retrograde endocannabinoid signaling, Oxytocin signaling pathway, and Ferroptosis pathways were altered in both low and high temperature attack groups. We hypothesize that IHNV alters gut microbes and body metabolism, leading to stress, inflammation, autoimmunity, and potential effects on body’s nervous system. The Fc epsilon RI signaling pathway, Neuroactive ligand-receptor interaction and Bile secretion are the pathways that changed in C vs. A but not in C vs. D. The Fc epsilon RI signaling pathway is an important immune-related pathway that plays an important function when the organism is infected by pathogenic bacteria (Chen et al., 2018). Neuroactive ligand-receptor interaction is directly related to neurological function (Wei et al., 2020). Bile has the ability to neutralize viruses and reduce viral infectivity (Lee et al., 1975). Pathways that were not changed in the C vs. A comparison but were changed in the C vs. D group included Apoptosis and the Sphingolipid signaling pathway. Apoptosis is an autonomous programmed cell death mechanism that helps maintain the stability of theacellular environment, allowing the body to eliminate aged and necrotic cells and maintain homeostasis. However, apoptotic cell numbers significantly increase in to stimuli-induced damage (Elmore, 2007). Inflammation can also trigger programmed cellular necrosis (Newton and Manning, 2016). The Sphingolipid signaling pathway is closely associated with carcinogenesis (Li et al., 2013). Sphingolipids are a class of natural metabolites that can be synthesized by both humans and symbiotic Bacteria can influence host health through sphingipid-like natural products (Hou et al., 2022). Differential metabolites in A vs. B are enriched in pathways related to immunity, antivirals, and other factors. The differential metabolites of C vs. D are primarily associated with organismal stability and immunity. Groups A and B were both infected with IHNV, which not only affected the host’s innate immunity and host homeostasis, but also the intestinal epithelial cells and intestinal microorganisms secreted some antiviral products in order to resist the damage caused by IHNV. However, the secretion of these antiviral products varied at different culture temperatures, and the differences in these metabolites related to antiviral activity may be closely associated with varying mortality rates of rainbow trout infected with IHNV at different temperatures. Groups C and D were not infected with IHNV, and the differences in temperature mainly affected the hosts’ innate immunity and homeostasis. Our study showed that the stability and immunity of the organism differed between high and low temperature farming, and that infection with IHNV at high and low-temperature altered the microbial metabolism of the gut and the metabolism of the organism, but there were differences between culture temperatures.

Many of the metabolites in the intestinal tract are of microbial origin and play a crucial role in maintaining the host’s health. Several differential metabolites associated with various bacteria are identified in comparisons between C and A, D and B, A and B, as well as C and D. Harmful bacteria associated with the selected differential metabolites in C vs. A include *Staphylococcus aureus*, *Vibrio vulnificus*, *Yersinia kristensenii* and *Streptococcus parauberis*. *Staphylococcus aureus* is a high-risk pathogen that can cause inflammatory responses and affect normal host physiology (Knox et al., 2015). *Vibrio vulnificus* is a widely distributed group of pathogens that can cause primary sepsis (Lu et al., 2023). *Yersinia* is distributed animals and has the ability to resist macrophage killing, which can lead to sepsis or localized infections in humans and animals (Joutsen et al., 2020). *Streptococcus parauberis* is widespread and can cause mass species (Kim et al., 2022). Interestingly, some metabolites of C vs. A also correlate with *Fomitopsis pinicola*, aorganism known for its antitumor, antioxidant, immune-enhancing, and anti-inflammatory effects (Lee et al., 2008; Bishop, 2020). Differential metabolites correlate with *Lactococcus raffinolactis* in D vs. B. Studies have shown that *lactic acid* has the ability to modulate host immunity, control homeostasis in the gut and resistance to pathogenic bacteria (Hagi and Hoshino, 2009). In A vs. B metabolites were correlated with *Hypsizygus marmoreus*, and the study indicated that *Hypsizygus marmoreus* has antioxidant capacity (Xu et al., 2020). The selected important metabolites are associated with several beneficial microorganisms, and these microorganisms and their metabolites may have potential as drugs against IHNV.

## 5. Conclusion

The selected important metabolites are associated with several beneficial microorganisms, and these microorganisms and their metabolites may have potential as drugs against IHNV. IHN causes gut microbial dysbiosis, with a significant increase in some conditionally pathogenic bacteria and a significant decrease in photosynthesis and carbon fixation by gut microbes. However, conditionally pathogenic bacteria increased more after infection with IHNV at low-temperature than after infection with IHNV at high-temperature. Additionally, metabolites in the gut also changed significantly after IHNV infection, and some immune-related metabolites differed between temperatures of IHNV infection. Finally, we screened probiotics *Fomitopsis pinicola*, *Lactococcus raffinolactis* and *Hypsizygus marmoreus* that may have the potential to prevent and control IHN. The results exposed the immune responses of rainbow trout gut microorganisms and metabolites to IHNV under different culture temperatures, and screened out the beneficial bacteria with potential effects against IHN, which provided a new idea and scientific basis for the prevention and treatment of IHN.

## Data availability statement

The datasets presented in this study can be found in online repositories. The names of the repository/repositories and accession number(s) can be found in the article/Supplementary material.

## Ethics statement

The animal study was approved by the Animal Care Committee of Gansu Agricultural University. The study was conducted in accordance with the local legislation and institutional requirements.

## Author contributions

QH and JW designed the study and wrote the initial manuscript. QH, JW, WK, SC, JL, NL, YL, ZLu, and ZLi prepared the samples and conducted the bioinformatics analysis. All authors contributed to the article and approved the submitted version.
