# Supplementary material for: Metagenomic and metabolomic analysis of changes in intestinal contents of rainbow trout (Oncorhynchus mykiss) infected with infectious hematopoietic necrosis virus at different culture water temperatures
Source: Front Microbiol. 2023 Oct 16;14:1275649. doi: 10.3389/fmicb.2023.1275649 (PMC10614001; doi:10.3389/fmicb.2023.1275649)
Supplement: Supplementary file 1 [file Data_Sheet_1.docx]

**
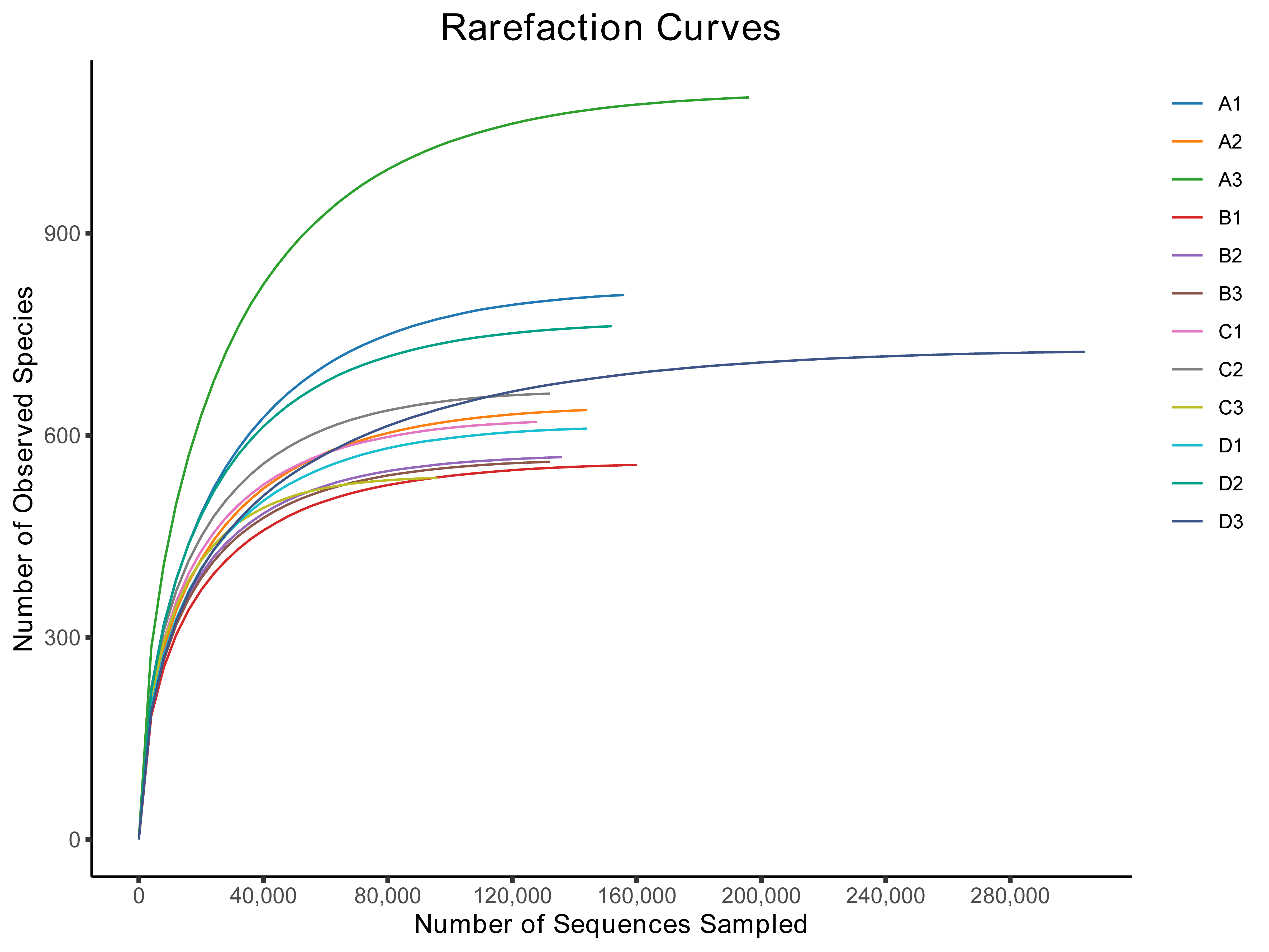
**

**Supplementary Figure 1.** All sample rarefaction curve

**
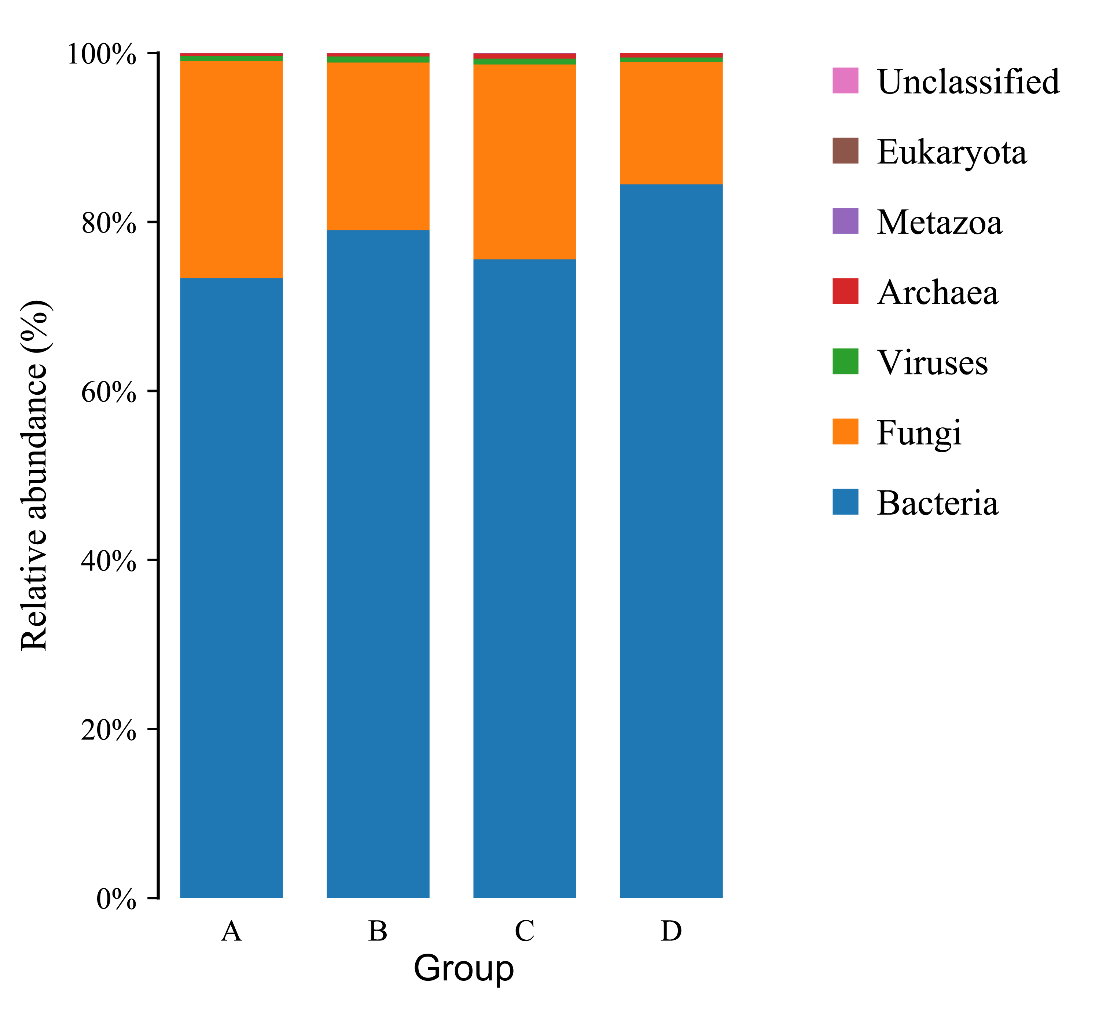
**

**Supplementary Figure 2.** Composition of the intestinal flora of each group at the kingdom level.

**
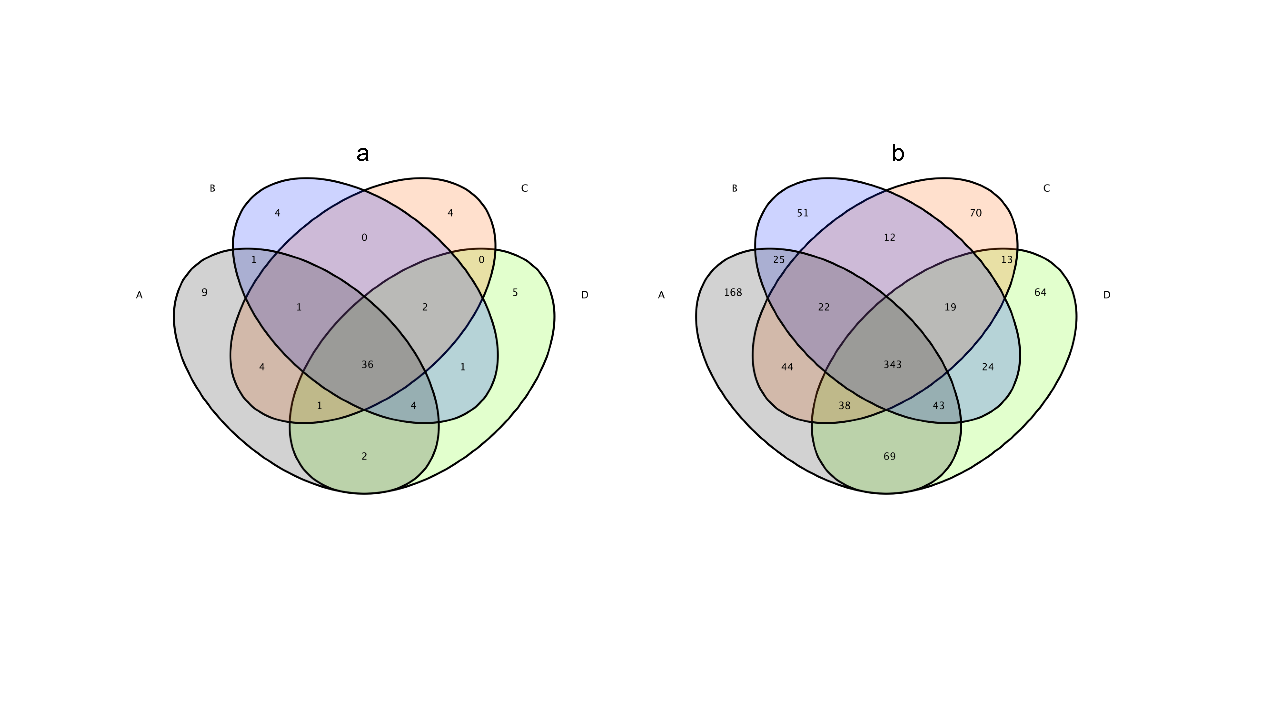
**

**Supplementary Figure 3.** (a) Phylum level Venn diagram. (b) Genus level Venn diagram.

**Supplementary Table 1** Composition of groups A and C at the phylum level

| ID | *P* | A(Mean) | A(Std) | C(Mean) | C(Std) |
| --- | --- | --- | --- | --- | --- |
| Proteobacteria | 0.560143 | 0.070208 | 0.031919 | 0.058111 | 0.008430 |
| Mucoromycota | 0.622685 | 0.012830 | 0.007677 | 0.010065 | 0.004690 |
| Firmicutes | 0.895458 | 0.010787 | 0.003839 | 0.011106 | 0.000914 |
| Basidiomycota | 0.144469 | 0.004951 | 0.000713 | 0.007673 | 0.002505 |
| Ascomycota | 0.570840 | 0.002280 | 0.002154 | 0.004288 | 0.005212 |
| Microsporidia | 0.313723 | 0.002212 | 0.001136 | 0.001386 | 0.000503 |
| Actinobacteria | 0.263694 | 0.000859 | 0.000423 | 0.001250 | 0.000307 |
| Aquificae | 0.129731 | 0.000833 | 0.000229 | 0.001144 | 0.000167 |
| Bacteroidetes | 0.395874 | 0.000739 | 0.000182 | 0.000592 | 0.000197 |
| Chytridiomycota | 0.801460 | 0.000734 | 0.000165 | 0.000708 | 0.000025 |
| Chlamydiae | 0.237872 | 0.000666 | 0.000406 | 0.001112 | 0.000382 |
| Zoopagomycota | 0.214864 | 0.000433 | 0.000186 | 0.000272 | 0.000029 |
| Candidatus Tectomicrobia | 0.220118 | 0.000343 | 0.000054 | 0.000395 | 0.000032 |
| Candidatus Heimdallarchaeota | 0.809883 | 0.000243 | 0.000020 | 0.000250 | 0.000043 |
| Cyanobacteria | 0.693409 | 0.000093 | 0.000040 | 0.000082 | 0.000024 |
| Acidobacteria | 0.889396 | 0.000072 | 0.000044 | 0.000067 | 0.000041 |
| Cryptomycota | 0.600361 | 0.000068 | 0.000013 | 0.000062 | 0.000012 |
| Euryarchaeota | 0.390362 | 0.000067 | 0.000010 | 0.000057 | 0.000013 |
| Arthropoda | 0.404975 | 0.000052 | 0.000007 | 0.000060 | 0.000013 |
| Blastocladiomycota | 0.425645 | 0.000042 | 0.000016 | 0.000052 | 0.000011 |
| Chloroflexi | 0.799801 | 0.000039 | 0.000011 | 0.000041 | 0.000011 |
| Planctomycetes | 0.988556 | 0.000031 | 0.000002 | 0.000030 | 0.000024 |
| Tenericutes | 0.363840 | 0.000024 | 0.000028 | 0.000008 | 0.000005 |
| Spirochaetes | 0.106714 | 0.000018 | 0.000010 | 0.000006 | 0.000004 |
| Candidatus Marinimicrobia | 0.841686 | 0.000012 | 0.000013 | 0.000010 | 0.000014 |
| Chlorobi | 0.837596 | 0.000010 | 0.000007 | 0.000009 | 0.000005 |
| Verrucomicrobia | 0.148045 | 0.000009 | 0.000010 | 0.000027 | 0.000014 |
| Crenarchaeota | 0.380830 | 0.000006 | 0.000006 | 0.000010 | 0.000002 |
| Candidatus Woesearchaeota | 0.552436 | 0.000005 | 0.000006 | 0.000003 | 0.000002 |
| Candidatus Curtissbacteria | 0.353432 | 0.000003 | 0.000003 | 0.000001 | 0.000001 |
| Gemmatimonadetes | 0.572799 | 0.000003 | 0.000003 | 0.000004 | 0.000004 |
| Candidatus Parcubacteria | 0.056989 | 0.000002 | 0.000001 | 0.000000 | 0.000000 |
| Candidatus_Rokubacteria | 0.941263 | 0.000002 | 0.000003 | 0.000002 | 0.000003 |
| Candidatus Nealsonbacteria | 0.780337 | 0.000002 | 0.000003 | 0.000003 | 0.000003 |
| Candidate division_Zixibacteria | 0.331525 | 0.000002 | 0.000001 | 0.000001 | 0.000001 |
| Candidatus Lindowbacteria | 0.625320 | 0.000002 | 0.000001 | 0.000002 | 0.000002 |
| Candidatus Doudnabacteria | 0.373901 | 0.000001 | 0.000002 | 0.000000 | 0.000000 |
| Candidatus Beckwithbacteria | 0.150301 | 0.000001 | 0.000001 | 0.000000 | 0.000000 |
| Fusobacteria | 0.373901 | 0.000001 | 0.000002 | 0.000000 | 0.000000 |
| Rhodothermaeota | 0.955594 | 0.000001 | 0.000002 | 0.000001 | 0.000002 |
| Candidate division WOR-3 | 0.840955 | 0.000001 | 0.000002 | 0.000001 | 0.000001 |
| Candidatus Blackburnbacteria | 0.373901 | 0.000001 | 0.000002 | 0.000000 | 0.000000 |
| Elusimicrobia | 0.373901 | 0.000001 | 0.000002 | 0.000000 | 0.000000 |
| Chordata | 0.982776 | 0.000001 | 0.000002 | 0.000001 | 0.000001 |
| Candidatus Hydrogenedentes | 0.373901 | 0.000001 | 0.000001 | 0.000000 | 0.000000 |
| Candidatus Microgenomates | 0.373901 | 0.000001 | 0.000001 | 0.000000 | 0.000000 |
| Candidatus Pacebacteria | 0.859544 | 0.000001 | 0.000001 | 0.000001 | 0.000002 |
| Thaumarchaeota | 0.373901 | 0.000001 | 0.000001 | 0.000000 | 0.000000 |
| Balneolaeota | 0.373901 | 0.000001 | 0.000001 | 0.000000 | 0.000000 |
| Candidatus Desantisbacteria | 0.373901 | 0.000000 | 0.000001 | 0.000000 | 0.000000 |
| Ignavibacteriae | 0.373901 | 0.000000 | 0.000001 | 0.000000 | 0.000000 |
| Nitrospirae | 0.373901 | 0.000000 | 0.000001 | 0.000000 | 0.000000 |
| Candidatus Bathyarchaeota | 0.373901 | 0.000000 | 0.000001 | 0.000000 | 0.000000 |
| Candidatus Melainabacteria | 0.373901 | 0.000000 | 0.000001 | 0.000000 | 0.000000 |
| Candidatus Peregrinibacteria | 0.373901 | 0.000000 | 0.000001 | 0.000000 | 0.000000 |
| Armatimonadetes | 0.666011 | 0.000000 | 0.000001 | 0.000001 | 0.000002 |
| Deinococcus-Thermus | 0.197070 | 0.000000 | 0.000000 | 0.000003 | 0.000003 |
| Candidatus Nomurabacteria | 0.373901 | 0.000000 | 0.000000 | 0.000000 | 0.000000 |
| Calditrichaeota | 0.116411 | 0.000000 | 0.000000 | 0.000004 | 0.000004 |
| Cnidaria | 0.154858 | 0.000000 | 0.000000 | 0.000004 | 0.000004 |
| Candidatus Latescibacteria | 0.373901 | 0.000000 | 0.000000 | 0.000001 | 0.000002 |
| Candidatus Cloacimonetes | 0.373901 | 0.000000 | 0.000000 | 0.000001 | 0.000002 |
| Candidatus Poribacteria | 0.373901 | 0.000000 | 0.000000 | 0.000001 | 0.000003 |
| Lentisphaerae | 0.373901 | 0.000000 | 0.000000 | 0.000001 | 0.000002 |

**Supplementary Table 1** Composition of groups B and D at the phylum level

| ID | *P* | B(Mean) | B(Std) | D(Mean) | D(Std) |
| --- | --- | --- | --- | --- | --- |
| Proteobacteria | 0.230438 | 0.057502 | 0.000959 | 0.121846 | 0.078847 |
| Firmicutes | 0.059448 | 0.033716 | 0.012880 | 0.013721 | 0.003199 |
| Mucoromycota | 0.122853 | 0.010730 | 0.001182 | 0.009119 | 0.000805 |
| Basidiomycota | 0.632797 | 0.004288 | 0.000659 | 0.004597 | 0.000797 |
| Microsporidia | 0.776600 | 0.002140 | 0.000467 | 0.001886 | 0.001370 |
| Ascomycota | 0.792259 | 0.001961 | 0.000961 | 0.001679 | 0.001450 |
| Aquificae | 0.646778 | 0.000939 | 0.000307 | 0.001084 | 0.000403 |
| Bacteroidetes | 0.754519 | 0.000819 | 0.000432 | 0.000730 | 0.000158 |
| Actinobacteria | 0.046372 | 0.000651 | 0.000032 | 0.000832 | 0.000105 |
| Chytridiomycota | 0.794157 | 0.000597 | 0.000052 | 0.000578 | 0.000107 |
| Zoopagomycota | 0.266568 | 0.000374 | 0.000074 | 0.000305 | 0.000056 |
| Chlamydiae | 0.597710 | 0.000348 | 0.000116 | 0.000462 | 0.000326 |
| Candidatus Tectomicrobia | 0.702923 | 0.000341 | 0.000029 | 0.000352 | 0.000033 |
| Candidatus Heimdallarchaeota | 0.050977 | 0.000219 | 0.000006 | 0.000291 | 0.000045 |
| Cyanobacteria | 0.939559 | 0.000103 | 0.000022 | 0.000105 | 0.000046 |
| Cryptomycota | 0.876644 | 0.000080 | 0.000039 | 0.000076 | 0.000021 |
| Euryarchaeota | 0.238587 | 0.000059 | 0.000019 | 0.000082 | 0.000021 |
| Arthropoda | 0.665979 | 0.000047 | 0.000023 | 0.000040 | 0.000017 |
| Blastocladiomycota | 0.699728 | 0.000038 | 0.000008 | 0.000042 | 0.000013 |
| Acidobacteria | 0.409469 | 0.000036 | 0.000024 | 0.000022 | 0.000009 |
| Spirochaetes | 0.535274 | 0.000033 | 0.000049 | 0.000014 | 0.000012 |
| Chloroflexi | 0.919398 | 0.000025 | 0.000003 | 0.000024 | 0.000013 |
| Planctomycetes | 0.966005 | 0.000023 | 0.000017 | 0.000023 | 0.000007 |
| Fusobacteria | 0.050546 | 0.000018 | 0.000010 | 0.000002 | 0.000003 |
| Candidatus Marinimicrobia | 0.274522 | 0.000011 | 0.000013 | 0.000001 | 0.000003 |
| Tenericutes | 0.164532 | 0.000011 | 0.000002 | 0.000006 | 0.000005 |
| Chlorobi | 0.057762 | 0.000010 | 0.000002 | 0.000007 | 0.000002 |
| Crenarchaeota | 0.756261 | 0.000007 | 0.000001 | 0.000010 | 0.000012 |
| Verrucomicrobia | 0.222269 | 0.000007 | 0.000005 | 0.000021 | 0.000016 |
| Thermodesulfobacteria | 0.373901 | 0.000006 | 0.000010 | 0.000000 | 0.000000 |
| Gemmatimonadetes | 0.794509 | 0.000004 | 0.000004 | 0.000006 | 0.000006 |
| Candidatus Beckwithbacteria | 0.347135 | 0.000003 | 0.000003 | 0.000001 | 0.000002 |
| Deinococcus-Thermus | 0.906966 | 0.000003 | 0.000003 | 0.000003 | 0.000000 |
| Candidatus Woesearchaeota | 0.770977 | 0.000003 | 0.000005 | 0.000004 | 0.000004 |
| Candidatus Nealsonbacteria | 0.889996 | 0.000003 | 0.000002 | 0.000003 | 0.000002 |
| Candidate division_WOR-3 | 0.122062 | 0.000003 | 0.000003 | 0.000000 | 0.000000 |
| Candidatus Rokubacteria | 0.167693 | 0.000003 | 0.000001 | 0.000001 | 0.000002 |
| Candidatus Magasanikbacteria | 0.373901 | 0.000002 | 0.000004 | 0.000000 | 0.000000 |
| Nitrospirae | 0.784576 | 0.000002 | 0.000003 | 0.000001 | 0.000002 |
| Candidate division Zixibacteria | 0.754319 | 0.000002 | 0.000003 | 0.000001 | 0.000002 |
| Candidatus Omnitrophica | 0.373901 | 0.000001 | 0.000003 | 0.000000 | 0.000000 |
| Cnidaria | 0.999724 | 0.000001 | 0.000002 | 0.000001 | 0.000002 |
| Candidatus_Peregrinibacteria | 0.373901 | 0.000001 | 0.000002 | 0.000000 | 0.000000 |
| Candidatus_Lindowbacteria | 0.706709 | 0.000001 | 0.000002 | 0.000002 | 0.000001 |
| Candidatus_Poribacteria | 0.342853 | 0.000001 | 0.000002 | 0.000004 | 0.000004 |
| Elusimicrobia | 0.999724 | 0.000001 | 0.000002 | 0.000001 | 0.000002 |
| Candidatus_Pacebacteria | 0.712390 | 0.000001 | 0.000001 | 0.000001 | 0.000003 |
| Fibrobacteres | 0.373901 | 0.000001 | 0.000001 | 0.000000 | 0.000000 |
| Candidatus Dadabacteria | 0.838975 | 0.000001 | 0.000000 | 0.000001 | 0.000001 |
| Candidatus_Kaiserbacteria | 0.373901 | 0.000000 | 0.000000 | 0.000000 | 0.000000 |
| Candidatus_Margulisbacteria | 0.373901 | 0.000000 | 0.000000 | 0.000000 | 0.000000 |
| Candidatus Handelsmanbacteria | 0.373901 | 0.000000 | 0.000000 | 0.000001 | 0.000002 |
| Thermotogae | 0.373901 | 0.000000 | 0.000000 | 0.000001 | 0.000002 |
| Candidatus Microgenomates | 0.373901 | 0.000000 | 0.000000 | 0.000001 | 0.000001 |
| Candidatus Curtissbacteria | 0.139864 | 0.000000 | 0.000000 | 0.000002 | 0.000001 |
| Platyhelminthes | 0.303744 | 0.000000 | 0.000000 | 0.000006 | 0.000008 |
| Ignavibacteriae | 0.217414 | 0.000000 | 0.000000 | 0.000003 | 0.000003 |

**Table S3** Composition of groups A and B at the phylum level

| ID | *P* | A(Mean) | A(Std) | B(Mean) | B(Std) |
| --- | --- | --- | --- | --- | --- |
| Proteobacteria | 0.528618 | 0.070208 | 0.031919 | 0.057502 | 0.000959 |
| Mucoromycota | 0.663971 | 0.012830 | 0.007677 | 0.010730 | 0.001182 |
| Firmicutes | 0.041763 | 0.010787 | 0.003839 | 0.033716 | 0.012880 |
| Basidiomycota | 0.302740 | 0.004951 | 0.000713 | 0.004288 | 0.000659 |
| Ascomycota | 0.826467 | 0.002280 | 0.002154 | 0.001961 | 0.000961 |
| Microsporidia | 0.923954 | 0.002212 | 0.001136 | 0.002140 | 0.000467 |
| Actinobacteria | 0.444165 | 0.000859 | 0.000423 | 0.000651 | 0.000032 |
| Aquificae | 0.657402 | 0.000833 | 0.000229 | 0.000939 | 0.000307 |
| Bacteroidetes | 0.783050 | 0.000739 | 0.000182 | 0.000819 | 0.000432 |
| Chytridiomycota | 0.241357 | 0.000734 | 0.000165 | 0.000597 | 0.000052 |
| Chlamydiae | 0.261489 | 0.000666 | 0.000406 | 0.000348 | 0.000116 |
| Zoopagomycota | 0.638053 | 0.000433 | 0.000186 | 0.000374 | 0.000074 |
| Candidatus Tectomicrobia | 0.971559 | 0.000343 | 0.000054 | 0.000341 | 0.000029 |
| Candidatus Heimdallarchaeota | 0.120421 | 0.000243 | 0.000020 | 0.000219 | 0.000006 |
| Cyanobacteria | 0.737861 | 0.000093 | 0.000040 | 0.000103 | 0.000022 |
| Acidobacteria | 0.277986 | 0.000072 | 0.000044 | 0.000036 | 0.000024 |
| Cryptomycota | 0.639033 | 0.000068 | 0.000013 | 0.000080 | 0.000039 |
| Euryarchaeota | 0.566578 | 0.000067 | 0.000010 | 0.000059 | 0.000019 |
| Arthropoda | 0.736165 | 0.000052 | 0.000007 | 0.000047 | 0.000023 |
| Blastocladiomycota | 0.708960 | 0.000042 | 0.000016 | 0.000038 | 0.000008 |
| Chloroflexi | 0.114966 | 0.000039 | 0.000011 | 0.000025 | 0.000003 |
| Planctomycetes | 0.493840 | 0.000031 | 0.000002 | 0.000023 | 0.000017 |
| Tenericutes | 0.448689 | 0.000024 | 0.000028 | 0.000011 | 0.000002 |
| Spirochaetes | 0.623047 | 0.000018 | 0.000010 | 0.000033 | 0.000049 |
| Candidatus Marinimicrobia | 0.939178 | 0.000012 | 0.000013 | 0.000011 | 0.000013 |
| Chlorobi | 0.989077 | 0.000010 | 0.000007 | 0.000010 | 0.000002 |
| Verrucomicrobia | 0.803198 | 0.000009 | 0.000010 | 0.000007 | 0.000005 |
| Crenarchaeota | 0.754471 | 0.000006 | 0.000006 | 0.000007 | 0.000001 |
| Candidatus Woesearchaeota | 0.727945 | 0.000005 | 0.000006 | 0.000003 | 0.000005 |
| Candidatus Curtissbacteria | 0.180266 | 0.000003 | 0.000003 | 0.000000 | 0.000000 |
| Gemmatimonadetes | 0.557917 | 0.000003 | 0.000003 | 0.000004 | 0.000004 |
| Candidatus Parcubacteria | 0.032914 | 0.000002 | 0.000001 | 0.000000 | 0.000000 |
| Candidatus Rokubacteria | 0.847334 | 0.000002 | 0.000003 | 0.000003 | 0.000001 |
| Candidatus Nealsonbacteria | 0.649196 | 0.000002 | 0.000003 | 0.000003 | 0.000002 |
| Candidate division Zixibacteria | 0.920541 | 0.000002 | 0.000001 | 0.000002 | 0.000003 |
| Candidatus Lindowbacteria | 0.726519 | 0.000002 | 0.000001 | 0.000001 | 0.000002 |
| Candidatus Doudnabacteria | 0.373901 | 0.000001 | 0.000002 | 0.000000 | 0.000000 |
| Candidatus Beckwithbacteria | 0.330655 | 0.000001 | 0.000001 | 0.000003 | 0.000003 |
| Fusobacteria | 0.042877 | 0.000001 | 0.000002 | 0.000018 | 0.000010 |
| Rhodothermaeota | 0.373901 | 0.000001 | 0.000002 | 0.000000 | 0.000000 |
| candidate_division_WOR-3 | 0.386735 | 0.000001 | 0.000002 | 0.000003 | 0.000003 |
| Candidatus Blackburnbacteria | 0.373901 | 0.000001 | 0.000002 | 0.000000 | 0.000000 |
| Elusimicrobia | 0.896163 | 0.000001 | 0.000002 | 0.000001 | 0.000002 |
| Chordata | 0.373901 | 0.000001 | 0.000002 | 0.000000 | 0.000000 |
| Candidatus_Hydrogenedentes | 0.373901 | 0.000001 | 0.000001 | 0.000000 | 0.000000 |
| Candidatus_Microgenomates | 0.373901 | 0.000001 | 0.000001 | 0.000000 | 0.000000 |
| Candidatus_Pacebacteria | 0.962975 | 0.000001 | 0.000001 | 0.000001 | 0.000001 |
| Thaumarchaeota | 0.373901 | 0.000001 | 0.000001 | 0.000000 | 0.000000 |
| Balneolaeota | 0.373901 | 0.000001 | 0.000001 | 0.000000 | 0.000000 |
| Candidatus_Desantisbacteria | 0.373901 | 0.000000 | 0.000001 | 0.000000 | 0.000000 |
| Nitrospirae | 0.496853 | 0.000000 | 0.000001 | 0.000002 | 0.000003 |
| Ignavibacteriae | 0.373901 | 0.000000 | 0.000001 | 0.000000 | 0.000000 |
| Candidatus_Bathyarchaeota | 0.373901 | 0.000000 | 0.000001 | 0.000000 | 0.000000 |
| Candidatus_Melainabacteria | 0.373901 | 0.000000 | 0.000001 | 0.000000 | 0.000000 |
| Armatimonadetes | 0.373901 | 0.000000 | 0.000001 | 0.000000 | 0.000000 |
| Candidatus_Peregrinibacteria | 0.583475 | 0.000000 | 0.000001 | 0.000001 | 0.000002 |
| Candidatus_Nomurabacteria | 0.373901 | 0.000000 | 0.000000 | 0.000000 | 0.000000 |
| Deinococcus-Thermus | 0.166988 | 0.000000 | 0.000000 | 0.000003 | 0.000003 |
| Candidatus_Omnitrophica | 0.373901 | 0.000000 | 0.000000 | 0.000001 | 0.000003 |
| Candidatus_Magasanikbacteria | 0.373901 | 0.000000 | 0.000000 | 0.000002 | 0.000004 |
| Candidatus_Poribacteria | 0.373901 | 0.000000 | 0.000000 | 0.000001 | 0.000002 |
| Fibrobacteres | 0.373901 | 0.000000 | 0.000000 | 0.000001 | 0.000001 |
| Candidatus Dadabacteria | 0.117010 | 0.000000 | 0.000000 | 0.000001 | 0.000000 |
| Thermodesulfobacteria | 0.373901 | 0.000000 | 0.000000 | 0.000006 | 0.000010 |
| Cnidaria | 0.373901 | 0.000000 | 0.000000 | 0.000001 | 0.000002 |

**Table S4** Composition of groups C and D at the phylum level

| ID | *P* | C(Mean) | C(Std) | D(Mean) | D(Std) |
| --- | --- | --- | --- | --- | --- |
| Proteobacteria | 0.236289 | 0.058111 | 0.008430 | 0.121846 | 0.078847 |
| Firmicutes | 0.244956 | 0.011106 | 0.000914 | 0.013721 | 0.003199 |
| Mucoromycota | 0.748059 | 0.010065 | 0.004690 | 0.009119 | 0.000805 |
| Basidiomycota | 0.112550 | 0.007673 | 0.002505 | 0.004597 | 0.000797 |
| Ascomycota | 0.450485 | 0.004288 | 0.005212 | 0.001679 | 0.001450 |
| Microsporidia | 0.584893 | 0.001386 | 0.000503 | 0.001886 | 0.001370 |
| Actinobacteria | 0.089145 | 0.001250 | 0.000307 | 0.000832 | 0.000105 |
| Aquificae | 0.821026 | 0.001144 | 0.000167 | 0.001084 | 0.000403 |
| Chlamydiae | 0.088306 | 0.001112 | 0.000382 | 0.000462 | 0.000326 |
| Chytridiomycota | 0.109888 | 0.000708 | 0.000025 | 0.000578 | 0.000107 |
| Bacteroidetes | 0.398051 | 0.000592 | 0.000197 | 0.000730 | 0.000158 |
| Candidatus Tectomicrobia | 0.176329 | 0.000395 | 0.000032 | 0.000352 | 0.000033 |
| Zoopagomycota | 0.419744 | 0.000272 | 0.000029 | 0.000305 | 0.000056 |
| Candidatus Heimdallarchaeota | 0.319834 | 0.000250 | 0.000043 | 0.000291 | 0.000045 |
| Cyanobacteria | 0.479595 | 0.000082 | 0.000024 | 0.000105 | 0.000046 |
| Acidobacteria | 0.132059 | 0.000067 | 0.000041 | 0.000022 | 0.000009 |
| Cryptomycota | 0.376872 | 0.000062 | 0.000012 | 0.000076 | 0.000021 |
| Arthropoda | 0.165188 | 0.000060 | 0.000013 | 0.000040 | 0.000017 |
| Euryarchaeota | 0.168924 | 0.000057 | 0.000013 | 0.000082 | 0.000021 |
| Blastocladiomycota | 0.341041 | 0.000052 | 0.000011 | 0.000042 | 0.000013 |
| Chloroflexi | 0.167765 | 0.000041 | 0.000011 | 0.000024 | 0.000013 |
| Planctomycetes | 0.617187 | 0.000030 | 0.000024 | 0.000023 | 0.000007 |
| Verrucomicrobia | 0.641544 | 0.000027 | 0.000014 | 0.000021 | 0.000016 |
| Candidatus Marinimicrobia | 0.358800 | 0.000010 | 0.000014 | 0.000001 | 0.000003 |
| Crenarchaeota | 0.998030 | 0.000010 | 0.000002 | 0.000010 | 0.000012 |
| Chlorobi | 0.472132 | 0.000009 | 0.000005 | 0.000007 | 0.000002 |
| Tenericutes | 0.647296 | 0.000008 | 0.000005 | 0.000006 | 0.000005 |
| Spirochaetes | 0.350527 | 0.000006 | 0.000004 | 0.000014 | 0.000012 |
| Gemmatimonadetes | 0.781082 | 0.000004 | 0.000004 | 0.000006 | 0.000006 |
| Calditrichaeota | 0.116411 | 0.000004 | 0.000004 | 0.000000 | 0.000000 |
| Cnidaria | 0.344203 | 0.000004 | 0.000004 | 0.000001 | 0.000002 |
| Candidatus_Nealsonbacteria | 0.772899 | 0.000003 | 0.000003 | 0.000003 | 0.000002 |
| Deinococcus-Thermus | 0.842807 | 0.000003 | 0.000003 | 0.000003 | 0.000000 |
| Candidatus_Woesearchaeota | 0.528481 | 0.000003 | 0.000002 | 0.000004 | 0.000004 |
| Candidatus_Rokubacteria | 0.440545 | 0.000002 | 0.000003 | 0.000001 | 0.000002 |
| Candidatus_Lindowbacteria | 0.647057 | 0.000002 | 0.000002 | 0.000002 | 0.000001 |
| Candidatus_Poribacteria | 0.455277 | 0.000001 | 0.000003 | 0.000004 | 0.000004 |
| Lentisphaerae | 0.373901 | 0.000001 | 0.000002 | 0.000000 | 0.000000 |
| Rhodothermaeota | 0.373901 | 0.000001 | 0.000002 | 0.000000 | 0.000000 |
| Candidatus_Pacebacteria | 0.865016 | 0.000001 | 0.000002 | 0.000001 | 0.000003 |
| Candidatus_Cloacimonetes | 0.373901 | 0.000001 | 0.000002 | 0.000000 | 0.000000 |
| candidate_division_Zixibacteria | 0.963257 | 0.000001 | 0.000001 | 0.000001 | 0.000002 |
| candidate_division_WOR-3 | 0.143244 | 0.000001 | 0.000001 | 0.000000 | 0.000000 |
| Candidatus_Latescibacteria | 0.373901 | 0.000001 | 0.000002 | 0.000000 | 0.000000 |
| Armatimonadetes | 0.373901 | 0.000001 | 0.000002 | 0.000000 | 0.000000 |
| Candidatus_Curtissbacteria | 0.581977 | 0.000001 | 0.000001 | 0.000002 | 0.000001 |
| Chordata | 0.373901 | 0.000001 | 0.000001 | 0.000000 | 0.000000 |
| Candidatus_Parcubacteria | 0.373901 | 0.000000 | 0.000000 | 0.000000 | 0.000000 |
| Candidatus_Kaiserbacteria | 0.373901 | 0.000000 | 0.000000 | 0.000000 | 0.000000 |
| Candidatus_Margulisbacteria | 0.373901 | 0.000000 | 0.000000 | 0.000000 | 0.000000 |
| Candidatus_Handelsmanbacteria | 0.373901 | 0.000000 | 0.000000 | 0.000001 | 0.000002 |
| Candidatus_Dadabacteria | 0.373901 | 0.000000 | 0.000000 | 0.000001 | 0.000001 |
| Thermotogae | 0.373901 | 0.000000 | 0.000000 | 0.000001 | 0.000002 |
| Elusimicrobia | 0.373901 | 0.000000 | 0.000000 | 0.000001 | 0.000002 |
| Candidatus_Microgenomates | 0.373901 | 0.000000 | 0.000000 | 0.000001 | 0.000001 |
| Nitrospirae | 0.373901 | 0.000000 | 0.000000 | 0.000001 | 0.000002 |
| Candidatus_Beckwithbacteria | 0.373901 | 0.000000 | 0.000000 | 0.000001 | 0.000002 |
| Ignavibacteriae | 0.217414 | 0.000000 | 0.000000 | 0.000003 | 0.000003 |
| Platyhelminthes | 0.303744 | 0.000000 | 0.000000 | 0.000006 | 0.000008 |
| Fusobacteria | 0.299510 | 0.000000 | 0.000000 | 0.000002 | 0.000003 |
